# Corrosion-Induced Degradation of Low-Cycle Fatigue Behavior in Q690qNH Weathering Steel: Coupling of Hierarchical Rust Layer Evolution and Surface Damage

**DOI:** 10.3390/ma19214524

**Published:** 2026-10-27

**Authors:** Yaohan Du, Mingzhe Liu, Huangjing Tao, Xuefeng Xiao, Qiuchi Xiao, Kun Yang, Xue Li, Hong Zhang, Ying Yang, Qingyuan Wang

**Affiliations:** 1 School of Architecture and Civil Engineering, Chengdu University, Chengdu 610106, China; duyaohan@cdu.edu.cn (Y.D.); lium07519@gmail.com (M.L.); t1026850568@gmail.com (H.T.); st332699@outlook.com (X.X.); 212026085901012@cdu.edu.cn (Q.X.); lixue@cdu.edu.cn (X.L.); 2 State Key Laboratory of Hydraulics and Mountain River Engineering, Sichuan University, Chengdu 610065, China; 3 Institute for Advanced Study, Chengdu University, Chengdu 610106, China; scu_yangkun@163.com; 4 Failure Mechanics and Engineering Disaster Prevention Key Laboratory of Sichuan Province, Sichuan University, Chengdu 610065, China; zzhanghong@scu.edu.cn; 5 Iron & Steel Research Institute of Ansteel Group Corporation, Anshan 114009, China; yying666@126.com

**Keywords:** weathering steel, low-cycle fatigue, salt spray corrosion, corrosion pits, rust layer evolution

## Abstract

**Highlights:**

- Hierarchical rust-layer reconstruction from γ-FeOOH to α-FeOOH/Fe_3_O_4_ promotes corrosion-rate stabilization.
- Corrosion-pit growth intensifies local stress concentration and cyclic softening, accelerating low-cycle fatigue degradation with exposure time.
- Corrosion switches crack initiation from microstructural heterogeneities to pits, while microstructure remains dominant during crack propagation.

**Abstract:**

Marine atmospheric corrosion can compromise the fatigue reliability of high-strength weathering steel structures, yet the coupled effects of protective rust-layer evolution and corrosion-pit damage on low-cycle fatigue behavior remain insufficiently understood. This study systematically investigates the degradation mechanism of low-cycle fatigue (LCF) performance in high-strength weathering steel Q690qNH under a simulated marine atmospheric environment, with particular emphasis on the coupling among hierarchical rust-layer evolution, corrosion-pit damage, and fatigue failure. The results show that the rust layer gradually evolves from an unstable γ-FeOOH dominated structure into a dense and stable α-FeOOH/Fe_3_O_4_ structure during corrosion. Accompanied by Cr enrichment in the inner rust layer, a protective inner rust-layer structure is developed, leading to a significant reduction in the corrosion rate. Meanwhile, corrosion pits formed during the early stage of exposure induce localized stress concentration and plastic strain accumulation under cyclic loading, thereby accelerating cyclic softening and significantly reducing fatigue life. At a strain amplitude of 0.8%, the fatigue life decreases by 31.9%, 55.2%, 60.5%, and 66.7% after 7, 30, 45, and 60 days of corrosion exposure, respectively. Given the limited replication, the reported fatigue-life reductions represent observed trends and require confirmation through additional testing. In uncorroded specimens, fatigue cracks mainly initiate at ferrite/M-A constituent interfaces, whereas corrosion pits become the dominant crack initiation sites after corrosion exposure; nevertheless, subsequent crack propagation remains governed by grain boundaries and microstructural characteristics. These results demonstrate that the LCF performance of Q690qNH weathering steel is jointly governed by the competing effects of corrosion-pit damage accumulation and the formation of a protective rust layer.

## 1. Introduction

The marine atmospheric environment, characterized by chloride deposition, wet–dry cycling, and complex loading conditions, can accelerate corrosion damage in bridge steel structures, thereby posing serious threats to their service safety and long-term reliability [1,2]. High-strength weathering steel Q690qNH exhibits enhanced corrosion resistance through the optimization of trace alloying elements, including Cu, Cr, Ni, and Mo. These alloying elements modify the cation selective properties and microstructural characteristics of the rust layer, facilitating the spontaneous formation of a dense and protective oxide layer under atmospheric conditions [3,4]. This protective rust layer effectively impedes the further penetration of corrosive species, such as oxygen, water, and Cl^−^, into the steel substrate, thereby reducing the corrosion rate and providing weathering steels with long-term self-protective capability [5]. Therefore, understanding the formation and evolution mechanisms of protective rust layers is of great significance for evaluating the long-term service performance of weathering steels, particularly their fatigue reliability.

The long-term corrosion resistance of weathering steels is closely associated with the formation of a multilayered rust structure during the corrosion process. Previous studies have demonstrated that unstable corrosion products, such as γ-FeOOH, are predominantly formed during the initial stage of corrosion. With prolonged exposure, these corrosion products gradually transform into more thermodynamically stable phases, including α-FeOOH and Fe_3_O_4_, resulting in the development of a hierarchical rust layer with distinct inner and outer regions [6,7]. Among these corrosion products, the compact α-FeOOH-rich inner rust layer can effectively reduce porosity and restrict the diffusion of corrosive species and is therefore considered a critical factor contributing to the long-term protective capability of weathering steels [8,9]. In addition, the enrichment of alloying elements such as Cr and Cu at the rust layer/substrate interface may promote the formation of stable oxides and enhance the structural stability of the rust layer [10,11]. However, the synergistic relationships among elemental redistribution, phase evolution, and microstructural characteristics during the development of protective rust layers in high-strength weathering steels remain insufficiently understood.

Despite the protective effects of rust layers, corrosion-induced surface defects generated during long-term service may still become critical sites for fatigue failure. In particular, under extreme service conditions such as earthquakes and strong winds, bridge structures experience low-cycle fatigue (LCF) loading accompanied by significant cyclic plastic deformation, making structural steels highly susceptible to corrosion-induced surface damage [12,13]. Pitting corrosion, one of the most common localized corrosion forms in marine environments, preferentially develops at locally active sites such as inclusions, grain boundaries, and surface defects [14,15,16]. Corrosion pits modify the surface morphology and local stress field and can induce localized stress concentration and plastic strain accumulation, thereby facilitating fatigue crack initiation [17]. Albrecht et al. [18] reported that corrosion pits can act as preferential sites for fatigue crack initiation and are critical contributors to fatigue degradation in steel structures. Cai et al. [12] found that, following exposure of Q690qE high-strength steel to a simulated marine industrial atmospheric environment, the accumulation of Cl^−^ at the bottoms of corrosion pits accelerated localized corrosion, while stress concentration promoted the initiation of corrosion fatigue microcracks. Xu et al. [17] combined three-dimensional corrosion morphology characterization with finite element analysis to demonstrate the significant effects of pit size and pit–pit interactions on fatigue crack initiation. Furthermore, Guo et al. [19] demonstrated that pre-corrosion led to a continuous reduction in the elastic modulus, yield strength, and ultimate tensile strength of high-strength steel welded joints with increasing corrosion exposure time. Numerous studies have further confirmed that the localized stress concentration and plastic damage accumulation induced by corrosion pits are major factors contributing to fatigue life reduction [20,21,22,23]. However, the protective effect arising from rust-layer evolution and the detrimental effect associated with corrosion-pit accumulation have rarely been considered simultaneously in corrosion-fatigue studies, resulting in an incomplete understanding of their competing roles in long-term fatigue degradation.

In this study, simulated marine atmospheric cyclic corrosion testing combined with strain-controlled low-cycle fatigue experiments was employed to systematically investigate the relationship between corrosion evolution and fatigue degradation in Q690qNH high-strength weathering steel. First, multiscale characterization techniques, including X-ray diffraction (XRD) and scanning electron microscopy coupled with energy-dispersive spectroscopy (SEM-EDS), were employed to characterize the phase composition, elemental distribution, and microstructural evolution of the rust layer during corrosion exposure, thereby elucidating the development of the protective rust layer. Subsequently, three-dimensional surface morphology characterization and strain-controlled low-cycle fatigue tests were performed to investigate the effects of corrosion-pit evolution on cyclic plastic deformation, fatigue life, and crack initiation behavior. Finally, fracture surface observations and electron backscatter diffraction (EBSD) analysis were conducted to clarify the transition in fatigue crack initiation mechanisms between uncorroded and corroded specimens. By integrating the investigation of rust-layer evolution with the characterization of corrosion-induced surface damage, this study provides new insights into the competing effects of rust-layer protection and corrosion-pit accumulation on LCF degradation and the transition in crack initiation mechanisms in Q690qNH weathering steel.

## 2. Materials and Methods

### 2.1. Material and Specimens

**Figure 1:**
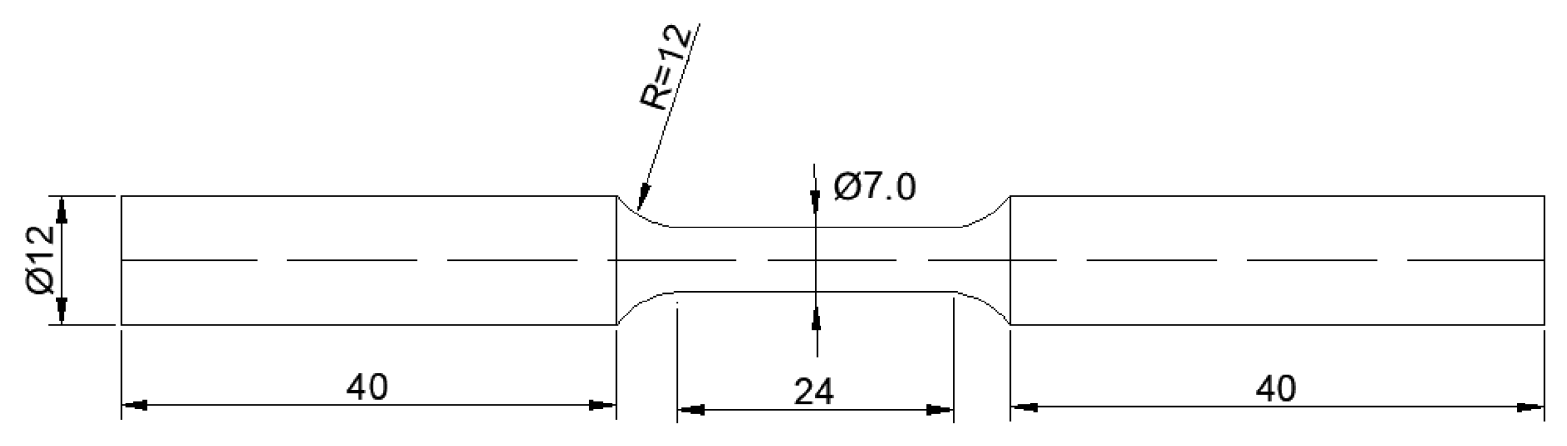
Specimen for fatigue test (unit: mm).

The experimental material used in this study was commercial high-strength weathering steel Q690qNH (Q: yield strength, q: bridge, NH: weather resistance, Anshan China), with the chemical compositions (wt%) provided in Table 1. The steel plates underwent hot rolling followed by tempering in accordance with the requirements for engineering bridge steels. Fatigue specimens were machined along the rolling direction of the plates, and their geometric dimensions are shown in Figure 1. Additionally, samples for corrosion characterization were prepared with dimensions are 50 mm in length, 25 mm in width, and 4 mm in height. All specimens were sequentially ground using SiC abrasive papers with grit sizes of 600, 800, 1200, and 2000, followed by mechanical polishing with silica suspensions containing particle sizes of 1.5, 1, 0.5, and 0.05 μm. Subsequently, the specimens were ultrasonically cleaned in anhydrous ethanol.

### 2.2. Accelerated Corrosion Experiment

Accelerated corrosion tests were conducted in a salt spray testing chamber (Gangpin Test equipment Co., Ltd., GP/SP-90P, Shanghai, China) using a modified cyclic corrosion procedure based on ISO 14993:2018 [24]. Each cycle lasted 8 h and consisted of 4 h of drying at 60 ± 1 °C and 25% relative humidity, followed by 4 h of salt spraying at 35 ± 1 °C and 95% relative humidity. The spray deposition rate was controlled at 1–2 mL/ (80 cm^2^·h), which was adjustable using glass nozzles. The salt solution was prepared by dissolving NaCl in distilled water to obtain a concentration of 50 g/L (5 wt%) at a pH of 7. During the cyclic corrosion test, the weight-loss specimens were placed at an approximate angle of 20° from the vertical direction, while fatigue specimens were suspended in the chamber to ensure uniform salt spray deposition on their surfaces. Both fatigue and weight-loss specimens were weighed before and after the corrosion test using a high-precision electronic balance (Sartorius, Göttingen, Germany, BSA224S; maximum capacity: 220 g; readability: 0.1 mg). The weight-loss test comprised six groups corresponding to corrosion exposure durations of 3, 7, 15, 30, 45, and 60 days, with four specimens for each exposure duration, including three specimens for corrosion mass-loss measurements and one specimen for microstructural characterization. For the fatigue tests, specimens were exposed to cyclic corrosion for 7, 30, 45, and 60 days, with two specimens tested at each exposure duration. A set of uncorroded specimens was also included as the control group for the fatigue tests. The corrosion products on the specimens designated for mass-loss measurements were removed by ultrasonic cleaning in a descaling solution consisting of HCl (500 mL), H_2_O (500 mL), and hexamethylenetetramine (3 g) [25].

### 2.3. Mechanical Test

#### 2.3.1. Low-Cycle Fatigue Test

The LCF tests were performed using a 50 kN electro-hydraulic servo fatigue testing machine (Shimadzu, EHFUV050K2-020-0A, Kyoto, Japan). The tests were conducted under strain control, with strain measured using a 12 mm axial extensometer (Epsilon, 3542-012M-010-LHT, Jackson, WY, USA). A triangular waveform with a strain ratio of R = −1 (fully reversed tension compression) was applied at a constant frequency of 0.5 Hz. Strain amplitudes of ±0.40%, ±0.60%, ±0.80%, ±1.00%, and ±1.20% were employed. For each strain amplitude, three uncorroded specimens and two corroded specimens were tested.

#### 2.3.2. Nanoindentation and Tensile Tests

The nanomechanical properties of the microstructure in Q690qNH steel were evaluated using a nanoindentation system (KLA iNano, Shanghai, China). Before indentation, the specimens were ground and polished, followed by metallographic etching with 4% nitric acid in alcohol to reveal the microstructural features. The tests were conducted in load-controlled mode with a maximum load of 50 mN. A Berkovich triangular-pyramidal indenter was used to quasi-statically indent the etched sample surface, with a loading time of 25 s and a dwell time of 10 s at the peak load. A total of 5 × 10 indentations were performed, with a spacing of 50 μm between indentations to avoid mutual interference. After indentation, the indentation marks were located using a SEM, and the effective indentations of three ferrite and M–A phases were determined, respectively. Then, the SEM observations were correlated with the corresponding indentation records to identify the indents associated with different microstructural constituents and determine their respective nanomechanical properties.

Tensile tests were performed using a universal testing machine (Shimadzu, AG-X Plus 100 kN, Kyoto, Japan). To investigate the effect of temperature on the tensile properties, tensile tests at room temperature and elevated temperature were conducted at the same strain rate of 5 × 10^−4^ s^−1^. In addition, to accurately measure the strain in the gauge section of the specimen, an extensometer was used during the tests to measure the specimen strain; it could also be used to precisely control the strain rate during tensile testing.

### 2.4. Finite Element Analysis

Finite element analysis (FEA) was performed using ABAQUS to investigate the stress concentration around corrosion pits under cyclic loading. The model was meshed using eight-node linear brick elements with reduced integration (C3D8R). The global seed size was 0.08 mm. Three corrosion pit radii of 51.68, 104.47, and 226.73 μm were considered, with corresponding local seed sizes of 0.010337, 0.020894, and 0.04 mm in the pit regions, respectively. Three peak pressure levels of 437, 674, and 875 MPa were considered for each pit geometry, resulting in nine loading cases. A Static, General analysis step was used, with geometric nonlinearity disabled. The pressure load was prescribed as a fully reversed triangular waveform with a stress ratio of (R = −1) over the normalized step-time interval of 0–1, representing one complete loading cycle. Stress fields were extracted from frames near the positive and negative pressure peaks, at step times of approximately 0.2475 and 0.7475, respectively. All nine cases reached the end of the analysis step (Step Time = 1.0), with 102 accepted increments per case and no cutbacks or severe discontinuities.

The material was modeled as Q690qNH steel, with an elastic modulus of 206 GPa and a Poisson’s ratio of 0.3. Plastic deformation was described using an isotropic hardening constitutive model, with the stress–strain data obtained from uniaxial tensile tests. Distributed coupling constraints were used to connect the loading surfaces at both ends of the specimen to reference points. One reference point was fully constrained, while the other was subjected to cyclic displacement-controlled loading.

### 2.5. Microstructural Characterization

Following salt spray corrosion exposure, the microstructural characterization specimens were preserved with the corrosion products intact on their surfaces. The specimens were then mounted, and their cross-sectional surfaces were ground using SiC abrasive paper up to 5000 grit, followed by mechanical polishing with a silica suspension. The cross-sectional morphology and elemental composition of the rust layer were characterized using a field-emission scanning electron microscope (FE-SEM, ZEISS, Sigma 300, Oberkochen, Germany) equipped with an energy-dispersive X-ray spectroscopy (EDS, Bruker X-MaxN 150) system. EDS elemental mapping was performed at an accelerating voltage of 20 kV, with an acquisition time of 20 min per elemental map. The distributions of Fe, O, Cl, Cr, Cu, Ni, and Mo were examined in representative cross-sectional regions of the rust layer. The acquired EDS spectra and elemental maps were processed using Bruker Esprit software 2.6. In addition, the three-dimensional morphology of corrosion pits on the specimen surfaces was characterized using a laser confocal 3D surface profilometer (IFM, Olympus, OLS51-SU, Tokyo, Japan). Then, the removed corrosion products were ground into a fine and homogeneous powder for phase analysis by X-ray diffraction (XRD, PANalytical Empyrean, Almelo, The Netherlands) using Co Kα radiation (λ = 1.7889 Å). The XRD patterns were collected by θ–2θ scanning over a 2θ range of 10–90° at a scanning rate of 0.02°/s.

After fatigue testing, the fracture morphologies of failed specimens were examined using FE-SEM. Subsequently, the specimens were mounted using copper powder and ground until the microstructure adjacent to the microcracks on the fracture surfaces was exposed. The mounted crack specimens were further ground using SiC paper up to 5000 grit and subsequently vibration-polished with a silica suspension. Finally, EBSD (TSL-DVC5) analysis was performed to investigate the crystallographic characteristics of corrosion-fatigue crack initiation and propagation. The measurements were conducted at an accelerating voltage of 20 kV and a working distance of 15 mm. The scanned area was 36 μm × 101 μm, with a step size of 0.5 μm and an indexing rate of 95%. EBSD data acquisition and post-processing were performed using OIM Analysis 7.3 to obtain crystallographic orientation maps, grain-boundary distribution maps, and kernel average misorientation (KAM) maps.

## 3. Results and Discussion

### 3.1. Microstructure and Mechanical Properties of Q690qNH Steel

**Figure 2:**
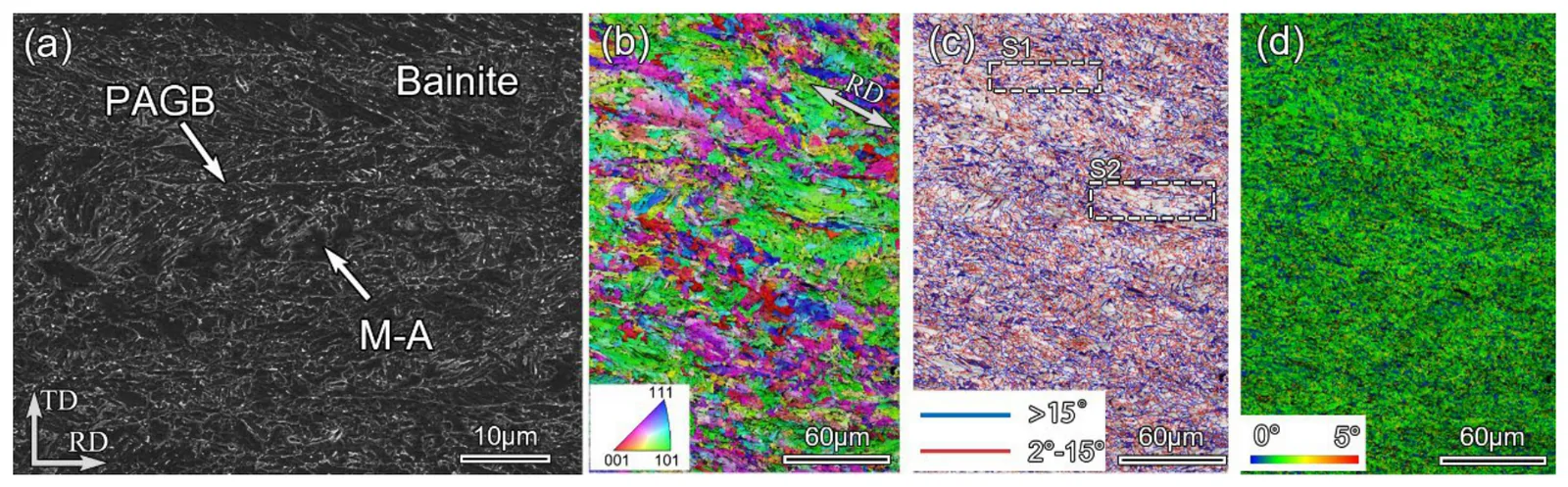
Microstructure of Q690qNH steel: (**a**) is the SEM image; (**b**) IPF + IQ; (**c**) IQ + GBs map; (**d**) the KAM plot.

Figure 2 presents the typical bainitic microstructure and crystallographic characteristics of the material. SEM observations reveal that the microstructure is predominantly composed of granular bainite, consisting of a ferritic matrix and blocky M-A constituents distributed along the grain boundaries. The bainitic regions exhibit an overall banded morphology along the rolling direction (RD) and are separated by prior austenite grain boundaries (PAGBs). EBSD analysis further reveals the crystallographic characteristics of the microstructure. As shown in Figure 2b, the inverse pole figure (IPF) map superimposed on the image quality (IQ) map indicates that some bainitic packets possess highly similar crystallographic orientations, forming bainitic clusters with similar orientation relationships. Further grain-boundary characterization (Figure 2c) shows that the interiors of the bainitic packets are mainly subdivided by low-angle grain boundaries (LAGBs, 2° < θ < 15°), whereas the acicular ferritic regions are primarily bounded by high-angle grain boundaries (HAGBs, θ > 15°), corresponding to regions S1 and S2, respectively. This multiscale grain-boundary structure reflects the crystallographic orientation relationships among different substructural units within the bainitic microstructure. In addition, the KAM results (Figure 2d) indicate that regions adjacent to the low-angle grain boundaries exhibit higher KAM values, suggesting more pronounced local lattice distortion and greater dislocation accumulation in these regions. This behavior is associated with the high-density dislocation structures generated during the bainitic transformation.

**Figure 3:**
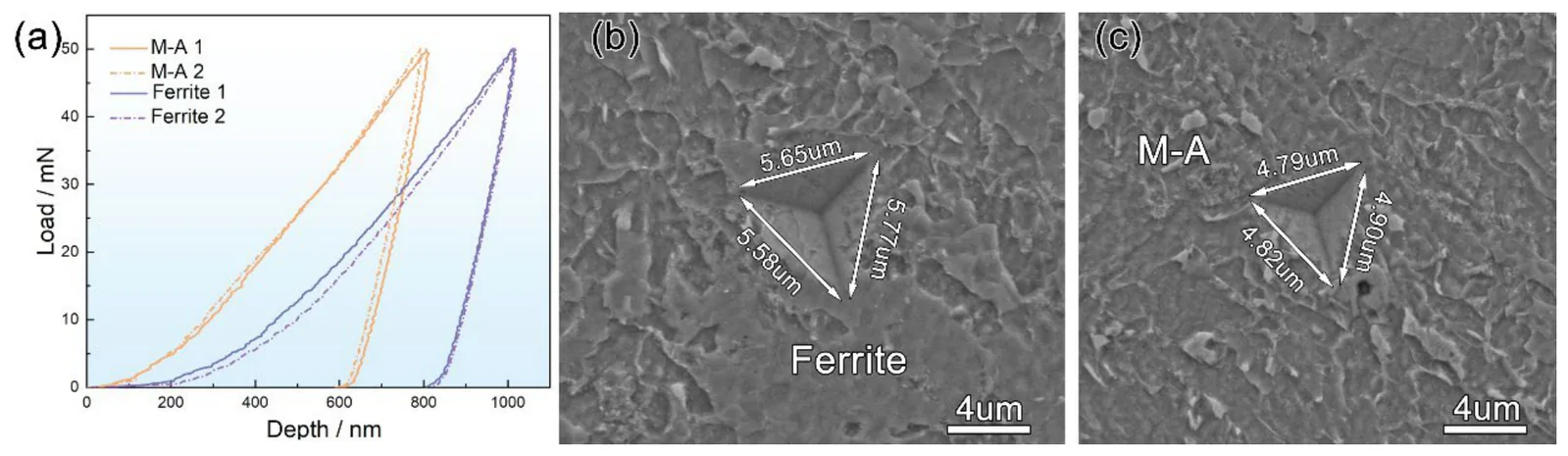
Nano-indentation tests on microstructures of the Q690qNH steel: (**a**) load–displacement curves, (**b**) indentation morphology on ferrite structure, (**c**) indentation morphology on M-A structure.

Nanoindentation testing was performed to characterize the localized nanomechanical behavior of different microstructural constituents in Q690qNH steel, providing further insight into the potential influence of microstructural heterogeneity on fatigue crack initiation. Figure 3a presents the repeated nanoindentation tests conducted on the M-A constituents and ferritic regions within the bainitic microstructure. The load–displacement curves reveal distinct mechanical responses between the two microstructural constituents. The M-A constituents exhibit a significantly higher nano-hardness of 4.1 ± 0.2 GPa, nearly twice that of the ferritic regions (2.4 ± 0.1 GPa). The corresponding residual indentation morphologies shown in Figure 3b,c exhibit more pronounced plastic deformation traces in the ferritic regions, further confirming their lower resistance to local plastic deformation.

This pronounced mechanical heterogeneity originates from the distinct mechanical properties of the hard M-A constituents and the soft ferritic matrix within the bainitic microstructure. The mismatch in elastic modulus and deformation capability between these two constituents can induce localized strain concentration near the M-A/ferrite interfaces, resulting in regions with high plastic strain gradients [26]. Such microscale mechanical heterogeneity may serve as potential active sites for fatigue crack initiation under cyclic loading, providing further evidence for understanding the fatigue crack initiation behavior of Q690qNH steel.

**Figure 4:**
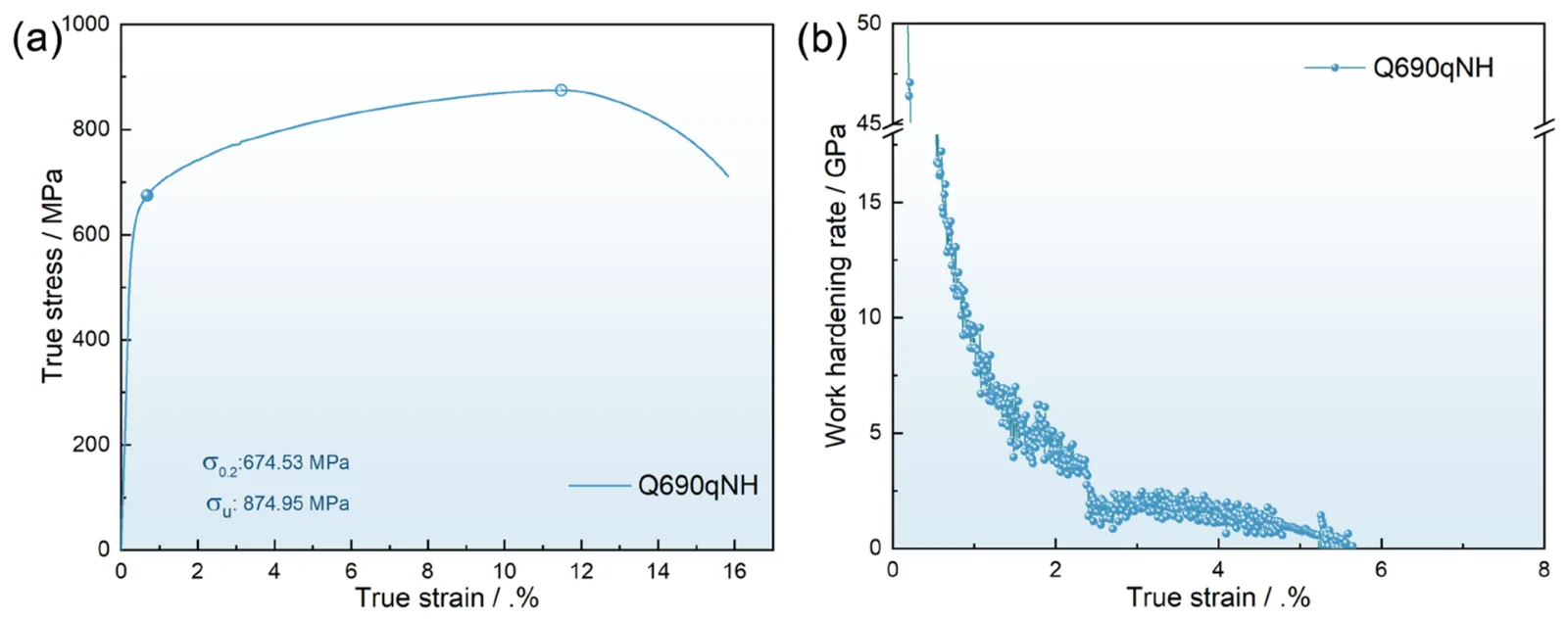
Monotonic behavior of Q690qNH: (**a**) True stress–strain curve; (**b**) instantaneous work hardening rate.

Figure 4 presents the monotonic tensile behavior and work-hardening characteristics of Q690qNH steel. According to the true stress-true strain curve (Figure 4a), the steel exhibits a high strength level, with a yield strength (σ_0.2_) of 674.53 MPa and an ultimate tensile strength (σ_u_) of 874.95 MPa. During the initial loading stage, the material undergoes predominantly elastic deformation. Once the applied stress exceeds the yield strength, plastic deformation initiates, accompanied by a pronounced strain-hardening response. With continued plastic straining, the true stress gradually increases and reaches its maximum value at a true strain of approximately 11%. Subsequently, the load-bearing capacity gradually decreases because of localized plastic deformation and damage accumulation, eventually leading to material failure and fracture. The corresponding evolution of the work-hardening rate is presented in Figure 4b. The Q690qNH steel exhibits a relatively high work-hardening capability during the early stage of plastic deformation. The work-hardening rate decreases rapidly with increasing plastic strain, indicating a pronounced strain-hardening response immediately after yielding. Subsequently, the work-hardening rate gradually declines and enters a relatively slower decreasing regime beyond a true strain of approximately 2%, indicating that the hardening capability progressively weakens with increasing plastic deformation. When the true strain exceeds approximately 5%, the work-hardening rate approaches a relatively low level, suggesting that the incremental strengthening capability of the material becomes increasingly limited. This behavior is consistent with the reduced stress increment observed during the later stage of tensile deformation.

The evolution of the work-hardening rate is governed by the competition among dislocation multiplication, impeded dislocation motion, and dislocation annihilation in different microstructural regions [27]. For the bainitic microstructure of Q690qNH steel, the refined bainitic structure, ferritic matrix, and mechanical heterogeneity between the M-A constituents and the surrounding matrix may further influence localized plastic deformation behavior, enabling the material to maintain a high strength level while retaining a certain degree of plastic deformability [28]. The relatively high yield strength indicates strong resistance to plastic deformation, whereas the gradual decrease in the work-hardening rate with increasing strain suggests that the plastic response under cyclic loading may be influenced by both the initial strengthening capability and the subsequent hardening stability of the material.

### 3.2. Evolution of Corrosion Products

#### 3.2.1. Corrosion Kinetics Behavior

**Figure 5:**
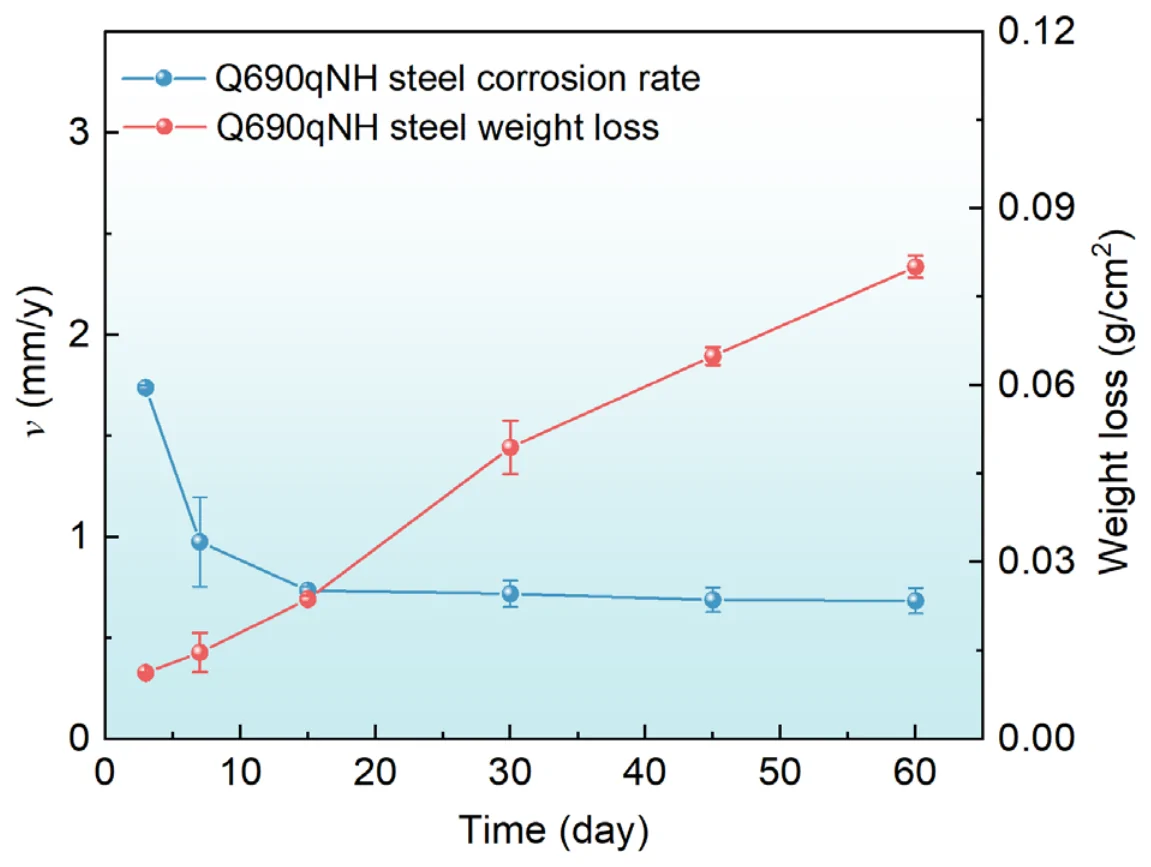
Weight loss and corrosion rate for Q690qNH steels after salt spray corrosion test.

Mass loss is an important parameter for evaluating the long-term corrosion behavior of steels. In this study, the corrosion kinetics of Q690qNH steel under cyclic corrosion conditions were investigated in accordance with ISO 9226:2012 (GB/T 19292.4-2018) [29]. The evolution of mass loss and corrosion rate was determined from the mass difference in the specimens before and after removal of the corrosion products. Figure 5 presents the corrosion kinetics behavior of Q690qNH steel at different corrosion durations, with the mass loss and average corrosion rate calculated from three parallel specimens.

With increasing corrosion time, the mass loss of the specimens increased continuously, indicating that corrosion reactions proceeded throughout the entire exposure period. However, the corrosion kinetics exhibited a transition from an initially active corrosion stage to a relatively stable corrosion regime. During the initial corrosion stage (≤3 days), the substrate surface was directly exposed to the corrosive environment, resulting in a relatively active corrosion process with an average corrosion rate of 1.73 ± 0.01 mm/y. With further extension of the corrosion time, the corrosion rate gradually decreased and continued to decline during the period of 3–7 days. After the corrosion time exceeded 15 days, the corrosion rate reached a relatively stable value of 0.68 ± 0.04 mm/y, representing a reduction of approximately 61% compared with that in the initial stage.

The continuous decrease in corrosion rate indicates that the corrosion process of Q690qNH steel gradually transitions from an active corrosion stage to a relatively stable stage. This evolution may be associated with the progressive formation and restructuring of rust layers during corrosion exposure. With increasing exposure time, corrosion products gradually cover the substrate surface and modify the interfacial interaction between the corrosive environment and the steel substrate, thereby influencing the subsequent corrosion kinetics [30]. To further clarify the rust evolution characteristics corresponding to the decrease in corrosion rate, various characterization techniques were employed to systematically investigate the evolution of corrosion products and the structure of the protective inner rust layer of Q690qNH steel.

#### 3.2.2. Phase Transformation and Element Redistribution

**Figure 6:**
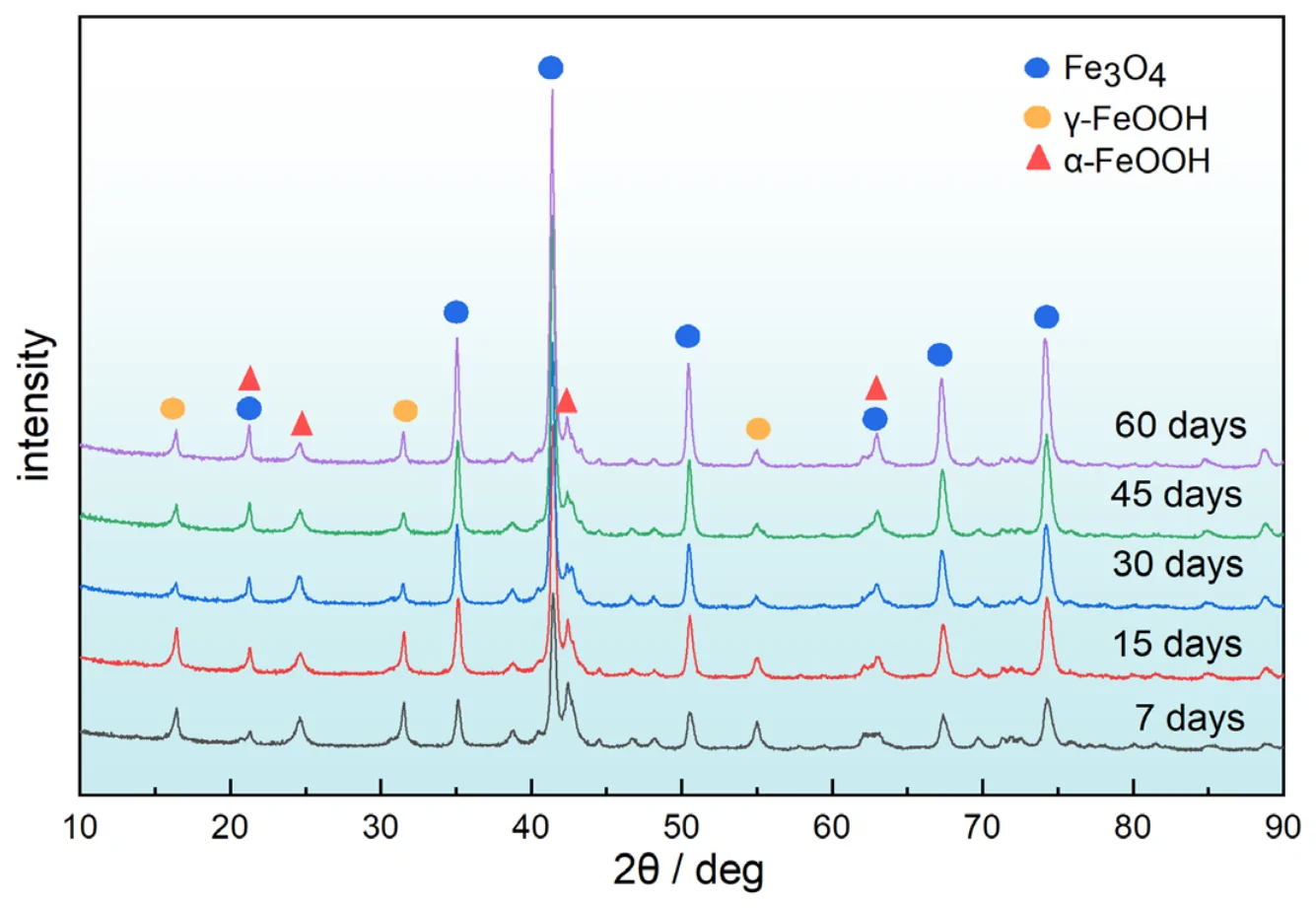
The phase compositions of the rust layer of Q690qNH steel after various days under corrosion.

Figure 6 presents the XRD patterns of rust products formed on Q690qNH steel after different corrosion durations, revealing the evolution of rust phase composition during corrosion exposure. According to the characteristic diffraction peaks, the corrosion products mainly consist of Fe_3_O_4_, α-FeOOH, and γ-FeOOH. A pronounced Fe_3_O_4_ (311) diffraction peak is observed at approximately 2θ = 41.37°, indicating the presence of crystalline magnetite in the rust layer. During the early corrosion stage (7–15 days), γ-FeOOH exhibits relatively strong diffraction peaks, suggesting that it is one of the dominant phases formed during the initial corrosion process. With increasing corrosion time, the diffraction intensity of γ-FeOOH gradually decreases, whereas the peaks associated with α-FeOOH and Fe_3_O_4_ become progressively stronger, indicating continuous evolution of the rust phase composition. These results suggest that the initially formed γ-FeOOH may undergo further transformation during prolonged corrosion exposure, accompanied by the development of more stable α-FeOOH and Fe_3_O_4_ phases.

Under the salt spray corrosion environment, the dissolution reprecipitation of γ-FeOOH, which is considered a metastable iron oxyhydroxide, may promote its transformation into more stable phases such as α-FeOOH [31]. Meanwhile, Fe_3_O_4_ is commonly observed in rust layers formed under marine atmospheric conditions and is generally located in the inner rust region near the substrate. Together with nanoscale α-FeOOH, Fe_3_O_4_ is considered to contribute to the formation of a compact and stable rust layer [32,33]. Therefore, the increased proportions of α-FeOOH and Fe_3_O_4_ accompanied by the decrease in γ-FeOOH with prolonged corrosion time indicates a gradual evolution of the rust layer toward a more stable phase composition, providing a basis for the development of a protective rust layer.

#### 3.2.3. Microstructure Evolution and Elemental Redistribution of Rust Layers

**Figure 7:**
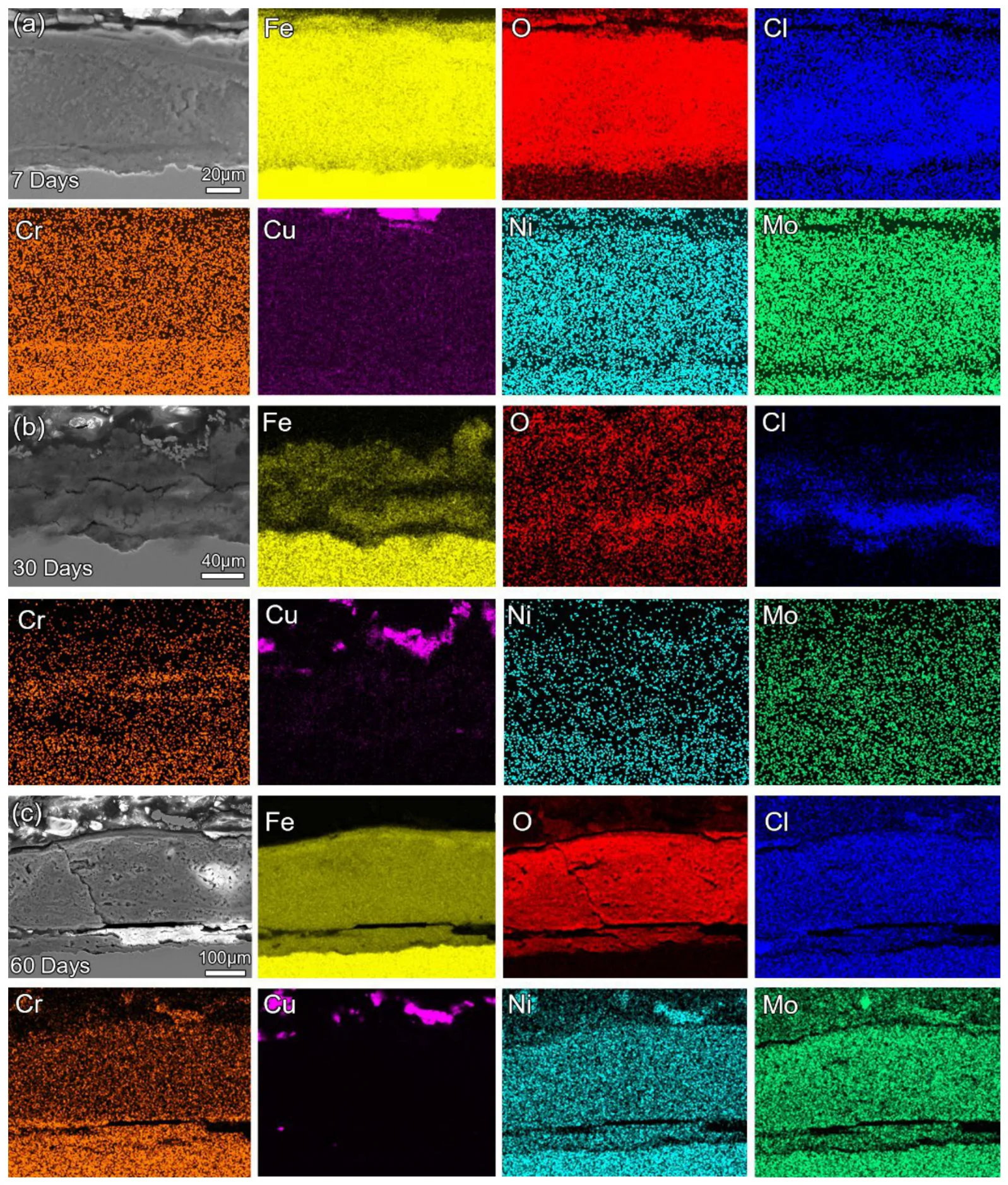
Cross-sectional morphology and element distribution of the rust layer formed after (**a**) 7, (**b**) 30 and (**c**) 60 days of exposure to corrosion environment.

To further elucidate the evolution of rust layer structures and the redistribution behavior of alloying elements during the corrosion process of Q690qNH steel, the cross-sectional morphologies and corresponding EDS elemental distributions of rust layers formed after 7, 30, and 60 days of salt spray corrosion were analyzed, as shown in Figure 7. After 7 days of corrosion exposure, the rust layer exhibited a relatively uniform morphology without an obvious layered structure. Fe and O were uniformly distributed throughout the rust layer, indicating that the corrosion products at this stage were mainly composed of iron oxides/oxyhydroxides. Meanwhile, no obvious localized enrichment of alloying elements, including Cr, Cu, Ni, and Mo, was observed, suggesting that significant redistribution of these elements had not yet occurred during the early corrosion stage. In contrast, Cl was distributed throughout the entire rust layer and preferentially accumulated in internal cracks and other defects within the rust layer, indicating that the initially formed rust layer was relatively loose and contained ion transport pathways that were insufficient to effectively hinder Cl^−^ migration toward the substrate.

With the extension of corrosion time to 30 days, the rust layer gradually thickened and began to exhibit more distinct layered characteristics. Meanwhile, changes in the spatial distributions of alloying elements were observed. Among these elements, Cr exhibited pronounced localized enrichment and formed continuous enrichment bands, indicating that Cr underwent preferential redistribution during corrosion exposure and gradually accumulated in specific regions of the rust layer. In contrast, no obvious enrichment of Cu, Ni, or Mo was observed, and their distributions remained relatively uniform. Meanwhile, the distribution of Cl changed from a relatively dispersed state at the early stage to localized accumulation, mainly concentrated in specific regions within the rust layer, suggesting that ion-transport behavior within the rust layer was influenced by the local structural characteristics as the corrosion process proceeded.

After 60 days of corrosion exposure, the rust layer further increased in thickness and developed a clearer inner–outer layered structure. The EDS results showed that Cr was further enriched in the inner rust region, while Cu, Ni, and Mo still exhibited certain redistribution characteristics. Notably, no obvious localized enrichment of Cl was detected in the mature rust layer, indicating that the stable rust structure formed during long-term corrosion exposure imposed a strong restriction on chloride ion transport. Combined with the aforementioned XRD results, the rust phase composition gradually evolved from relatively unstable iron oxyhydroxides at the early stage toward more stable phases such as α-FeOOH and Fe_3_O_4_ with increasing corrosion time, accompanied by alloying element redistribution and structural adjustment of the rust layer. These results indicate that long-term corrosion exposure promotes continuous reconstruction of the Q690qNH steel rust layer, transforming the initially defective corrosion products into a stable layered rust structure, thereby providing a structural basis for the subsequent formation of a protective inner rust layer.

Based on the above characterization results, the Q690qNH steel does not form a homogeneous corrosion product layer during long-term corrosion exposure. Instead, the corrosion products gradually evolve into a hierarchical rust layer with distinct compositional and structural gradients. During the initial corrosion stage, the outer rust layer is mainly composed of γ-FeOOH. With prolonged corrosion exposure, alloying elements such as Cr migrate toward and enrich in the inner rust region, accompanied by the development of stable α-FeOOH/Fe_3_O_4_ phases and progressive densification of the rust layer [10]. Therefore, the reduction in corrosion rate of Q690qNH steel is mainly attributed to the continuous evolution of the protective rust layer rather than merely to the coverage effect of corrosion products. In this process, the Cr-enriched region is associated with the formation of stable corrosion products, while the inner α-FeOOH/Fe_3_O_4_ phases act as the primary barrier to the transport of corrosive species, jointly restricting the migration of Cl^−^ toward the substrate and thereby contributing to long-term corrosion resistance [34,35]. However, the formation of a protective rust layer mainly inhibits the further penetration of corrosive media and cannot completely eliminate the localized surface defects generated during the early corrosion stage. Therefore, the initiation and evolution of corrosion pits remain critical factors governing subsequent fatigue damage behavior.

### 3.3. Corrosion-Induced Surface Damage Evolution

**Figure 8:**
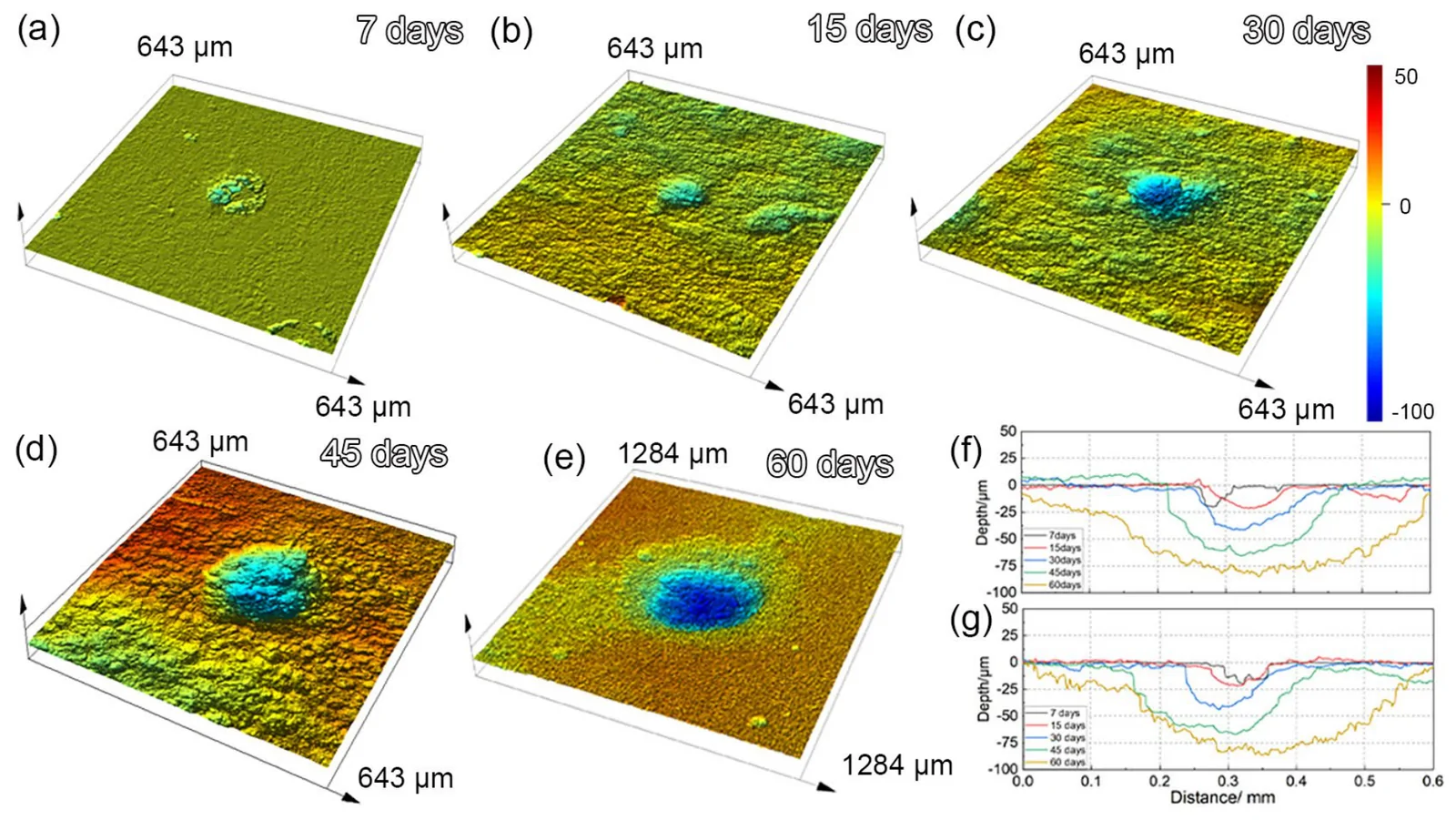
The 3D morphology of pitting of Q690qNH steel after different time of corrosion. (**a**) 7 days; (**b**) 15 days; (**c**) 30 days; (**d**) 45 days; (**e**) 60 days; (**f**) and (**g**) transverse and longitudinal profiles of corrosion pits.

To clarify the influence of corrosion-induced surface defects on the evolution of low-cycle fatigue damage in Q690qNH steel, the three-dimensional surface morphologies of corroded specimens after rust removal were characterized using laser confocal microscopy. The representative corrosion pit morphologies at each corrosion time are shown in Figure 8. With increasing cyclic corrosion duration, corrosion pits gradually initiated, grew, and coalesced, resulting in a continuous increase in surface roughness. After 7 days of corrosion exposure, distinct localized corrosion pits had already formed on the specimen surface, with a maximum depth of approximately 23 μm, whereas the surrounding areas remained relatively intact. With prolonged corrosion exposure, progressive pit growth and coalescence led to a transition from isolated defects to interconnected corrosion-damaged regions. After 30 days of corrosion exposure, the pit depth increased to approximately 43 μm, accompanied by pronounced surface fluctuations. After further exposure for 45 and 60 days, the dimensions of the corrosion pits increased significantly, with the pit depth and radius reaching approximately 71 μm and 141 μm, and 83 μm and 299 μm, respectively. These results indicate that prolonged corrosion exposure promoted the continuous expansion of localized corrosion regions and progressive deterioration of the surface morphology [36,37].

The formation of corrosion pits not only disrupts the geometrical continuity of the material surface but also induces local stress concentration under cyclic loading, thereby affecting fatigue crack initiation behavior [38]. The stress concentration factor *K_t_* of corrosion pits under *R* = −1 loading conditions can be calculated using the following equation:

(1) Kt=1+2dr

where *d* represents the corrosion-pit depth (mm) and *r* represents the corrosion-pit radius (mm). Using a laser confocal microscope, three-dimensional scans were conducted on multiple representative corrosion pits on the surface. The maximum, minimum and average values of the depth and radius of the 20 corrosion pits on the sample surface under different corrosion times were obtained, and the stress concentration factors *K_t_* under different corrosion conditions were calculated using the average values, as summarized in Table 2. The results show that *K_t_* gradually increased with increasing corrosion duration, indicating that the enlargement of corrosion pits and the evolution of more complex pit morphologies intensified the local stress concentration effect.

**Figure 9:**
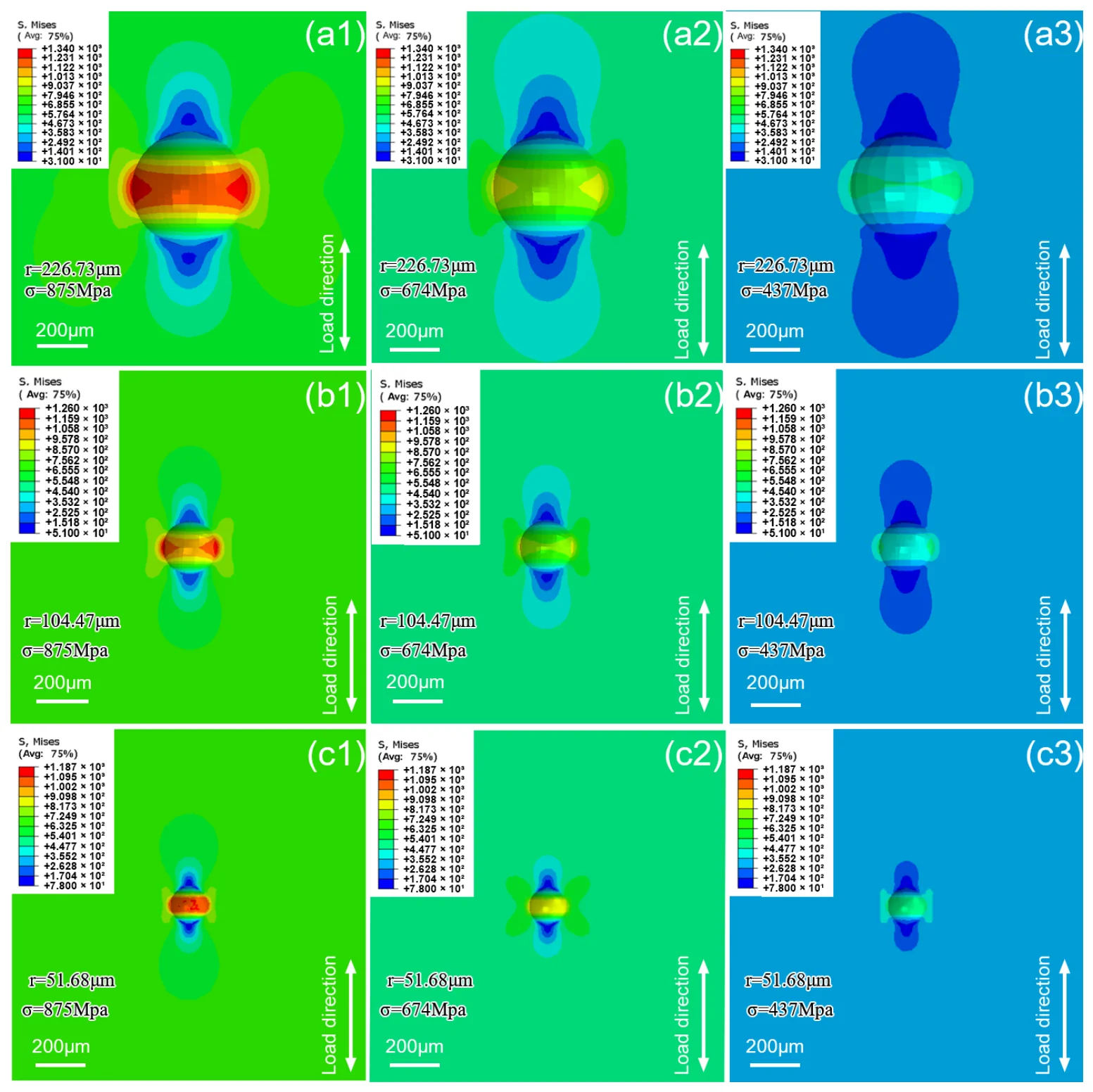
The comparison of stress distribution nephogram with different size parameters in the corrosion pits. (**a1**–**a3**) 60 days; (**b1**–**b3**) 30 days; (**c1**–**c3**) 7 days.

As shown in Figure 9, finite element simulations were performed using ABAQUS to analyze the local stress distribution around the idealized corrosion pit models with average sizes after 7 days, 30 days, and 60 days of corrosion pits with different geometrical dimensions under cyclic loading conditions. The results show that pronounced high-stress regions were observed at the bottom of corrosion pits, with the maximum stress concentrated near the pit edges. These results demonstrate that corrosion pits act as geometrical discontinuities that significantly amplify local stress levels. With increasing pit size, the high-stress regions further expanded, indicating that larger corrosion defects formed during long-term corrosion exposure are more likely to induce localized plastic deformation.

Therefore, the stress concentration generated at the bottoms of corrosion pits may promote local plastic strain accumulation and increase the likelihood of fatigue crack initiation. Particularly under cyclic tensile-compressive loading, the regions surrounding corrosion pits are prone to developing high dislocation densities, thereby accelerating heterogeneous deformation within the ferrite/bainite microstructure and promoting fatigue damage evolution [39]. Consequently, corrosion-induced surface geometrical defects may accelerate crack initiation and reduce fatigue life by altering the local stress state and plastic deformation behavior, thereby providing the structural basis for understanding the subsequent degradation of cyclic plasticity and fatigue resistance.

It should be noted that the analytical stress concentration factor *K_t_*, should be regarded as an idealized estimate based on simplified corrosion-pit geometry. The equation characterizes pit geometry using pit depth and radius and therefore cannot fully capture the irregular three-dimensional morphology of actual corrosion pits. Consequently, this simplified geometric approximation may not accurately represent the local stress field around irregular corrosion defects. Despite these limitations, the calculated *K_t_* values remain useful for a preliminary assessment of the effect of corrosion duration on geometric stress concentration. The finite element results shown in Figure 9 provide complementary evidence for this trend. The stress contours indicate that larger corrosion pits are associated with higher local stress levels and more extensive high-stress regions around the pit bottoms and edges. Therefore, the analytical and finite element results are consistent in terms of their qualitative trends.

### 3.4. Effect of Corrosion Damage on Cyclic Plastic Deformation Behavior

#### 3.4.1. Hysteresis Response

**Figure 10:**
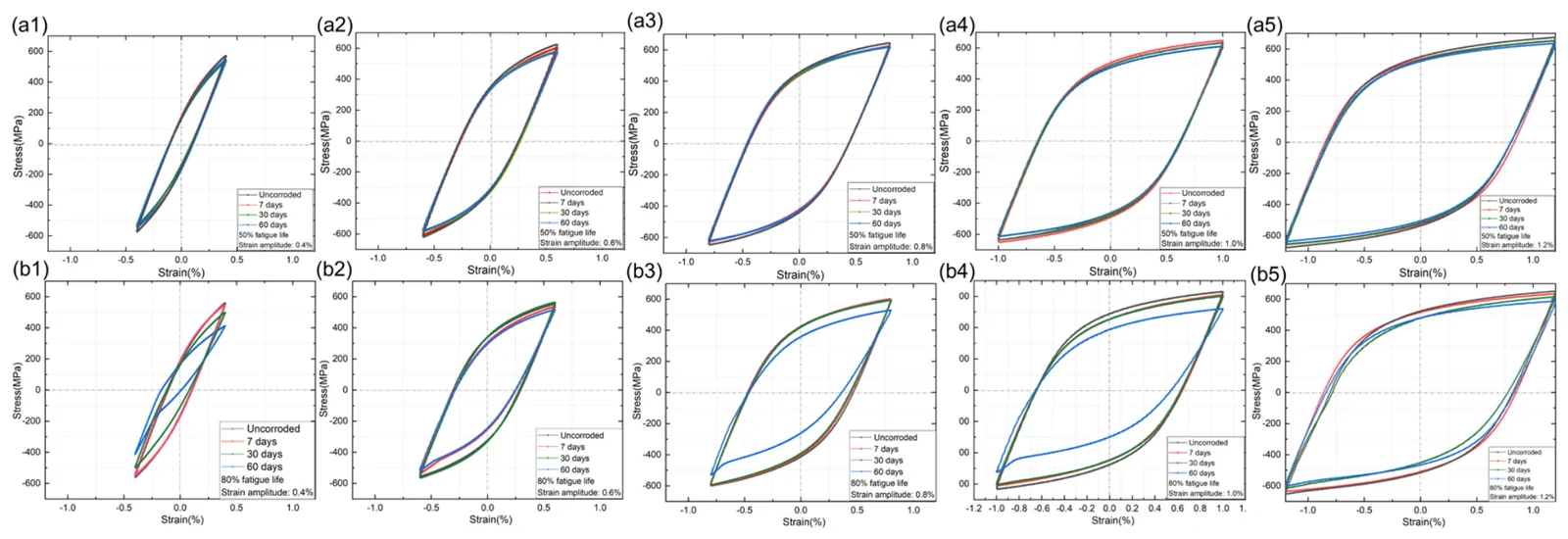
(**a1–a5**) The hysteresis loops curves of Q690qNH steel under 50% fatigue life at various corrosion times with corresponding strain amplitudes of (**a1**) 0.4%, (**a2**) 0.6%, (**a3**) 0.8%, (**a4**) 1% and (**a5**) 1.2%, respectively; (**b1–b5**) the hysteresis loops curves of Q690qNH steel under 80% fatigue life in corresponding strain amplitudes of (**b1**) 0.4%, (**b2**) 0.6%, (**b3**) 0.8%, (**b4**) 1% and (**b5**) 1.2%, respectively.

Figure 10(a1–a5) presents the stable cyclic stress–strain hysteresis responses of Q690qNH steel under different corrosion conditions and strain amplitudes at the half-life stage. The stable hysteresis loops were determined at half of the fatigue life (*N_f_*/2). It can be observed that the hysteresis-loop shape varies significantly with increasing applied strain amplitude for all specimens. At lower strain amplitudes, the hysteresis loops remain relatively narrow, indicating a smaller range of plastic strain developed during cyclic loading. With increasing strain amplitude, both the width and height of the hysteresis loops increase simultaneously, reflecting the involvement of greater plastic deformation in the cyclic response. The uncorroded Q690qNH steel exhibits relatively complete and stable hysteresis loops, indicating good cyclic plastic deformation capability and energy dissipation capacity. After corrosion exposure for different durations, the overall hysteresis-loop shapes remain largely unchanged; however, both the stress amplitude and loop area exhibit a slight decreasing trend with increasing corrosion duration. In particular, the hysteresis response of the specimens corroded for 7 days is comparable to that of the uncorroded specimens, suggesting that the relatively limited surface damage formed during the early corrosion stage has only a minor effect on the cyclic plastic response. With prolonged corrosion exposure to 30 and 60 days, the hysteresis-loop area gradually decreases, indicating that corrosion damage may reduce the cyclic load-bearing capacity and weaken the plastic energy dissipation per cycle.

Figure 10(b1–b5) further compares the cyclic hysteresis behavior of specimens under different corrosion conditions at 80% of the fatigue life. For all specimens, the hysteresis-loop area increases with increasing total strain amplitude, indicating that higher strain levels promote greater plastic deformation accumulation. Meanwhile, under the same strain amplitude, the stress amplitude at 80% of the fatigue life is lower than that at the half-life stage, suggesting that Q690qNH steel exhibits a certain degree of cyclic softening during fatigue cycling. For the uncorroded and 7-day corroded specimens, the hysteresis loop morphology remains relatively stable at 80% fatigue life. However, after corrosion exposure for 30 days, slight pinching behavior appears at higher strain amplitudes, indicating increased non-uniformity in plastic deformation during cyclic loading. After 60 days of corrosion exposure, pronounced hysteresis loop contraction is observed at nearly all strain amplitudes, suggesting that long-term corrosion damage significantly affects cyclic plastic stability [40]. This behavior is associated with surface defects formed during prolonged corrosion exposure. With increasing corrosion duration, the number and size of corrosion pits increase, generating local stress concentrations and promoting localized plastic strain accumulation under cyclic loading, thereby reducing the overall deformation compatibility of the material [41].

**Figure 11:**
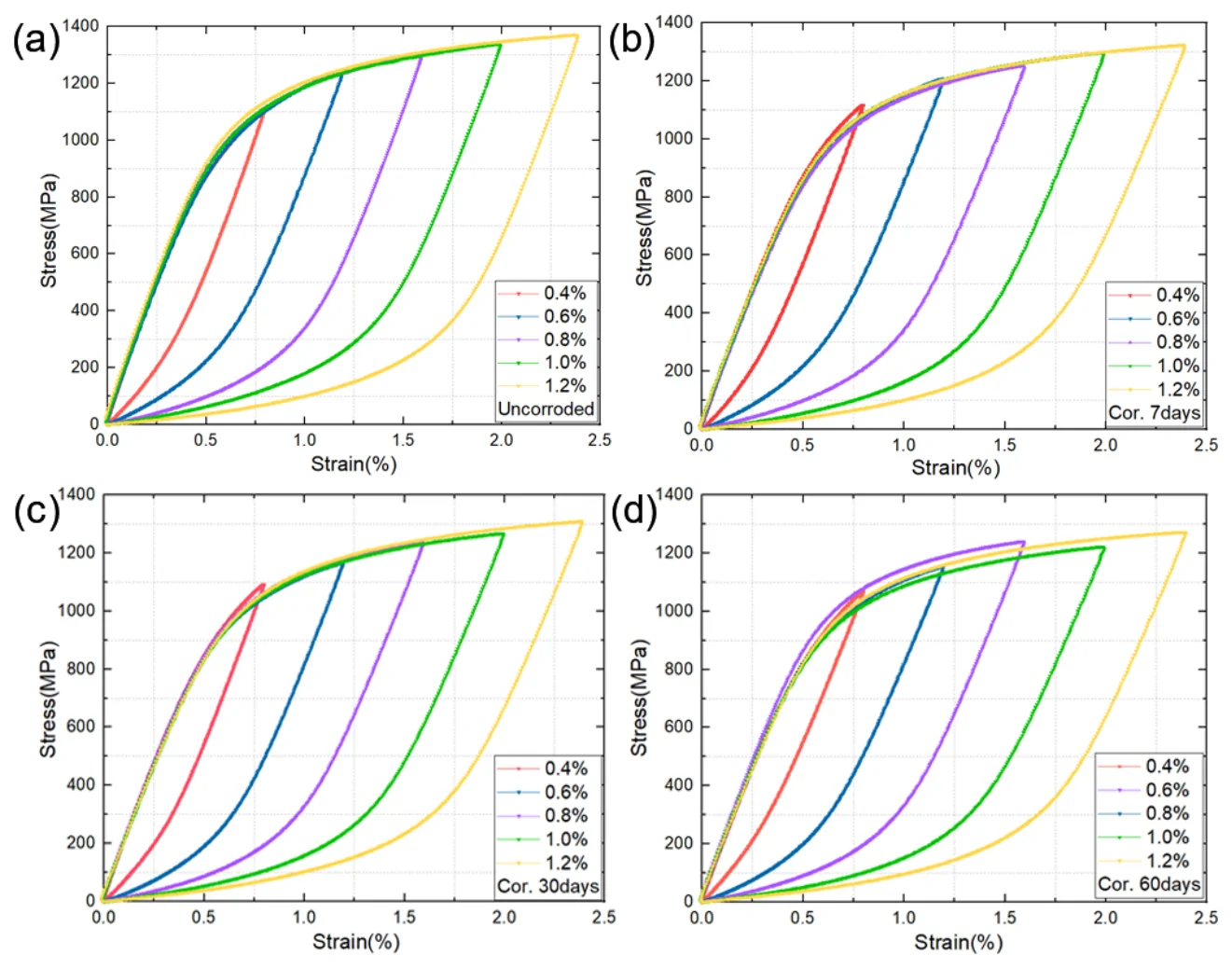
Masing behavior of smooth and corroded Q690qNH steel.

Figure 11a–d illustrate the Masing behavior of Q690qNH steel under different corrosion conditions. For the uncorroded specimens, the loading and unloading curves exhibit good self-similarity at different strain amplitudes, indicating that the cyclic plastic response follows the Masing relationship and reflects relatively homogeneous deformation mechanisms within the material. With increasing corrosion duration, the Masing behavior gradually deviates from the ideal relationship. In particular, after 60 days of corrosion exposure, although the elastic regions of the loading curves remain well overlapped, significant deviations appear in the inelastic deformation regions. The loading and unloading branches can no longer be superimposed through simple proportional scaling, indicating reduced stability of the cyclic plastic deformation behavior. The degradation of the Masing characteristics may be associated with corrosion-induced surface heterogeneity. The randomly distributed corrosion pits generated during long-term salt spray exposure can act as local stress concentration sites, promoting localized plastic strain accumulation during cyclic loading. This results in heterogeneous deformation responses in different regions and consequently disrupts the original self-similar cyclic deformation behavior of the material [42,43].

#### 3.4.2. Cyclic Softening Behavior

**Figure 12:**
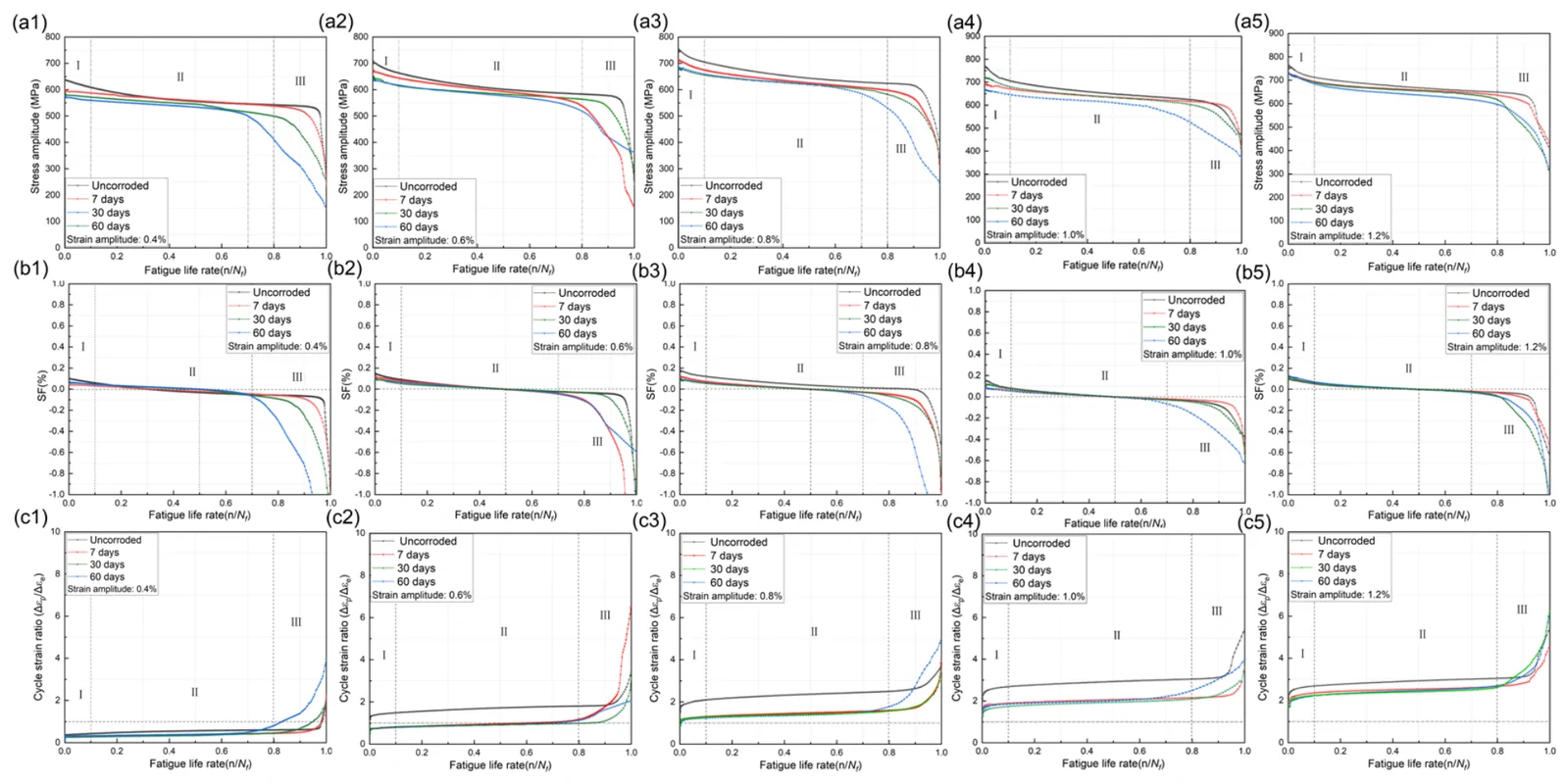
(**a1**–**a5**) The cyclic stress amplitude response of Q690qNH steel with corresponding strain amplitudes of (**a1**) 0.4%, (**a2**) 0.6%, (**a3**) 0.8%, (**a4**) 1% and (**a5**) 1.2%, respectively; (**b1**–**b5**) the cyclic softening factor (SF) curves of Q690qNH steel in corresponding strain amplitudes of (**b1**) 0.4%, (**b2**) 0.6%, (**b3**) 0.8%, (**b4**) 1% and (**b5**) 1.2%, respectively; (**c1**–**c5**) the cyclic strain ratio (SR) curves of Q690qNH steel in corresponding strain amplitudes of (**c1**) 0.4%, (**c2**) 0.6%, (**c3**) 0.8%, (**c4**) 1% and (**c5**) 1.2%, respectively.

The cyclic stress response curves reflect the evolution of the cyclic load-bearing capacity of Q690qNH steel during low-cycle fatigue. As shown in Figure 12(a1–a5), all specimens exhibit a continuous decrease in stress amplitude under different strain amplitudes, indicating pronounced cyclic softening behavior of Q690qNH steel during cyclic loading. With increasing strain amplitude, the initial stress amplitude increases, which can be attributed to the greater resistance to plastic deformation associated with larger cyclic strains. Under the same strain amplitude, the corroded specimens exhibit lower stress amplitudes than the uncorroded specimens, and the stress amplitude further decreases with increasing corrosion duration, indicating that prolonged corrosion exposure reduces the cyclic load-bearing capacity of the material.

According to the evolution of stress amplitude, the cyclic response can be divided into three stages. Stage I corresponds to the initial fatigue stage (0–10% *N_f_*), during which all specimens exhibit a rapid decrease in stress amplitude. This stage is mainly associated with dislocation rearrangement, internal strain accommodation, and the initial cyclic softening process during cyclic plastic deformation. Stage II covers approximately 10–80% of the fatigue life, during which the rate of stress-amplitude reduction decreases significantly and gradually stabilizes, indicating that the material enters a relatively stable cyclic deformation stage. Stage III occurs during the later stage of fatigue life, when the stress amplitude decreases rapidly, reflecting the accumulation of cyclic damage and enhanced localized plastic deformation, eventually leading to fatigue failure.

It is noteworthy that the corrosion duration significantly affects the onset of Stage III. The uncorroded and 7-day corroded specimens generally enter the rapid stress reduction stage after approximately 80% of fatigue life, whereas the specimens corroded for 30 and 60 days enter this stage earlier, at approximately 70% of fatigue life. This accelerated softening behavior may be associated with the non-uniform corrosion pits formed during prolonged corrosion exposure. These irregular corrosion pits can act as local stress concentration sites, inducing instability in inelastic deformation and promoting localized plastic strain accumulation under cyclic loading.

To further quantitatively characterize the cyclic softening behavior, the SF was introduced, and its calculation is given by Equation (2) [44]:

(2) $$\mathrm{SF}=\frac{\Delta \sigma \;-\Delta \sigma_{N_{f/2}}}{\Delta \sigma }$$

where Δ*σ* is the stress range of whole cyclic life and Δ*σ_Nf_*_/2_ is the stress range of half cyclic life. As shown in Figure 12(b1–b5), all specimens exhibit similar three-stage evolution characteristics in the SF curves. During the early fatigue stage, the SF decreases rapidly, indicating a rapid adjustment of the cyclic load-bearing capacity of the material. Subsequently, the SF enters a relatively stable stage with a lower rate of variation, suggesting that the internal mechanisms governing cyclic deformation reach a relatively stable state. In the later fatigue stage, the SF decreases rapidly again, corresponding to pronounced cyclic softening. Compared with the uncorroded specimens, the corroded specimens, particularly those exposed for 30 and 60 days, exhibit an earlier onset of SF reduction and a more pronounced decrease, indicating that corrosion damage enhances the cyclic softening behavior. This phenomenon may be related to corrosion-induced surface defects. With increasing corrosion duration, the size and number of corrosion pits increase, making local regions more susceptible to plastic strain concentration and thereby accelerating cyclic damage evolution.

Furthermore, plastic strain is a critical component of low-cycle fatigue behavior. To investigate the influence of corrosion on the elastic–plastic strain response of the studied steel, the SR, defined as the ratio of plastic strain amplitude to elastic strain amplitude, was introduced, and its calculation is given by Equation (3) [45]:

(3) $$\mathrm{SR}=\frac{\Delta \varepsilon_{P}}{\Delta \varepsilon_{e}}=\frac{E\Delta \varepsilon_{P}}{\Delta \sigma }=\frac{E\Delta \varepsilon }{\Delta \sigma }-1$$

where Δ*σ* is the stress range; Δ*ε*, Δ*ε_e_*, and Δ*ε_p_* are total strain range, elastic strain range, and plastic strain range, respectively. The evolution of SR is shown in Figure 12(c1–c5). During the early fatigue stage (Stage I), the SR of all specimens increases rapidly and subsequently enters a relatively stable stage (Stage II). The differences among specimens with different corrosion conditions are relatively small during this stage, indicating that early corrosion damage has a limited effect on the overall cyclic strain partitioning behavior. Upon entering the later fatigue stage (Stage III), the SR increases rapidly, indicating an increased contribution of plastic strain and the development of more pronounced non-uniform plastic deformation within the material. Compared with the uncorroded specimens, the specimens corroded for 60 days enter the rapid SR-increase stage earlier, particularly after approximately 70% of the fatigue life. This indicates that prolonged corrosion damage reduces the cyclic deformation stability of the material and promotes plastic strain localization [23,46].

#### 3.4.3. Low-Cycle Fatigue Life Degradation

The strain-life behavior of Q690qNH steel under different corrosion conditions was analyzed under strain-controlled loading conditions (*R* = −1) by combining the Basquin and Coffin-Manson models. The corresponding equations are given as follows:

(4) $$\frac{\Delta \varepsilon }{2}=\frac{\Delta \varepsilon_{e}}{2}+\frac{\Delta \varepsilon_{p}}{2}=\frac{\sigma_{f}^{\prime }}{E}(2N_{f}{)}^{b}+\varepsilon_{f}^{\prime }(2N_{f}{)}^{c}$$

where Δ*ε*, Δ*ε_e_*, and Δ*ε_p_* represent the strain range, elastic strain range, and plastic strain range, respectively; *σ_f_’* is the fatigue strength coefficient; *ε_f_’* is the fatigue ductility coefficient; b and c are the fatigue strength and ductility exponents, respectively; E is the elastic modulus; and *N_f_* is the fatigue life. Table 3 presents the fatigue life of the failed specimens of the material under different corrosion times and different strain amplitudes. Meanwhile, the fitting parameters for different corrosion days are shown in Table 4. It should be noted that only two corroded specimens were tested at each strain amplitude. This limited replication is insufficient to fully characterize the statistical variability of fatigue life. Therefore, the fitted parameters and calculated fatigue-life reductions should be interpreted as descriptive results for the tested specimens rather than statistically conclusive estimates.

**Figure 13:**
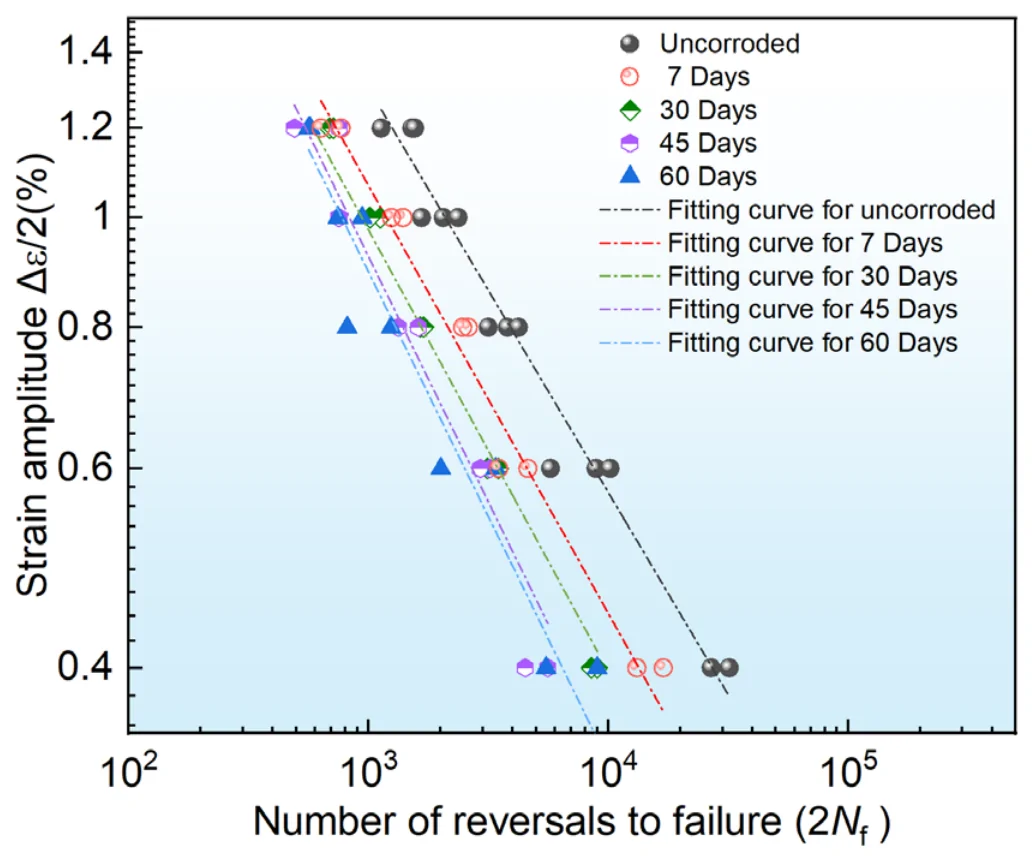
Relationship between strain amplitude and fatigue life of Q690qNH steel.

The scatter pattern and the fitted curve are shown in Figure 13, the fatigue life of all specimens increases with decreasing total strain amplitude, exhibiting typical strain-controlled fatigue characteristics. Under the same strain amplitude, the fatigue life of the corroded specimens is lower than that of the uncorroded specimens and further decreases with increasing corrosion time, indicating that surface damage induced by salt-spray corrosion has a significant effect on the fatigue performance of Q690qNH steel. At a relatively high strain amplitude of 1.2%, all specimens retain relatively long fatigue lives, demonstrating the good cyclic plastic deformation capability of Q690qNH steel. At a strain amplitude of 0.8%, compared with the reference sample without corrosion, the fatigue lives of specimens corroded for 7, 30, 45, and 60 days decrease by approximately 31.9%, 55.2%, 60.5%, and 66.7%, respectively. Among them, the fatigue life reduction after 60 days of corrosion was 6.2% greater than that after 45 days, indicating the deceleration in fatigue performance degradation during prolonged corrosion exposure.

This trend is consistent with the previously discussed corrosion kinetics and rust-layer evolution. Therefore, the reduction in the fatigue life of Q690qNH steel with increasing corrosion time is mainly associated with surface defects and localized stress concentrations generated during the early corrosion stage, whereas the protective gradient rust layer formed during prolonged corrosion exposure limits further corrosion progression, resulting in a gradual decrease in the rate of fatigue performance degradation. Consequently, the long-term fatigue performance of Q690qNH steel under corrosive environments is governed by the competing effects of corrosion damage accumulation and protective rust-layer formation, while the steel maintains relatively stable cyclic deformation capability.

**Figure 14:**
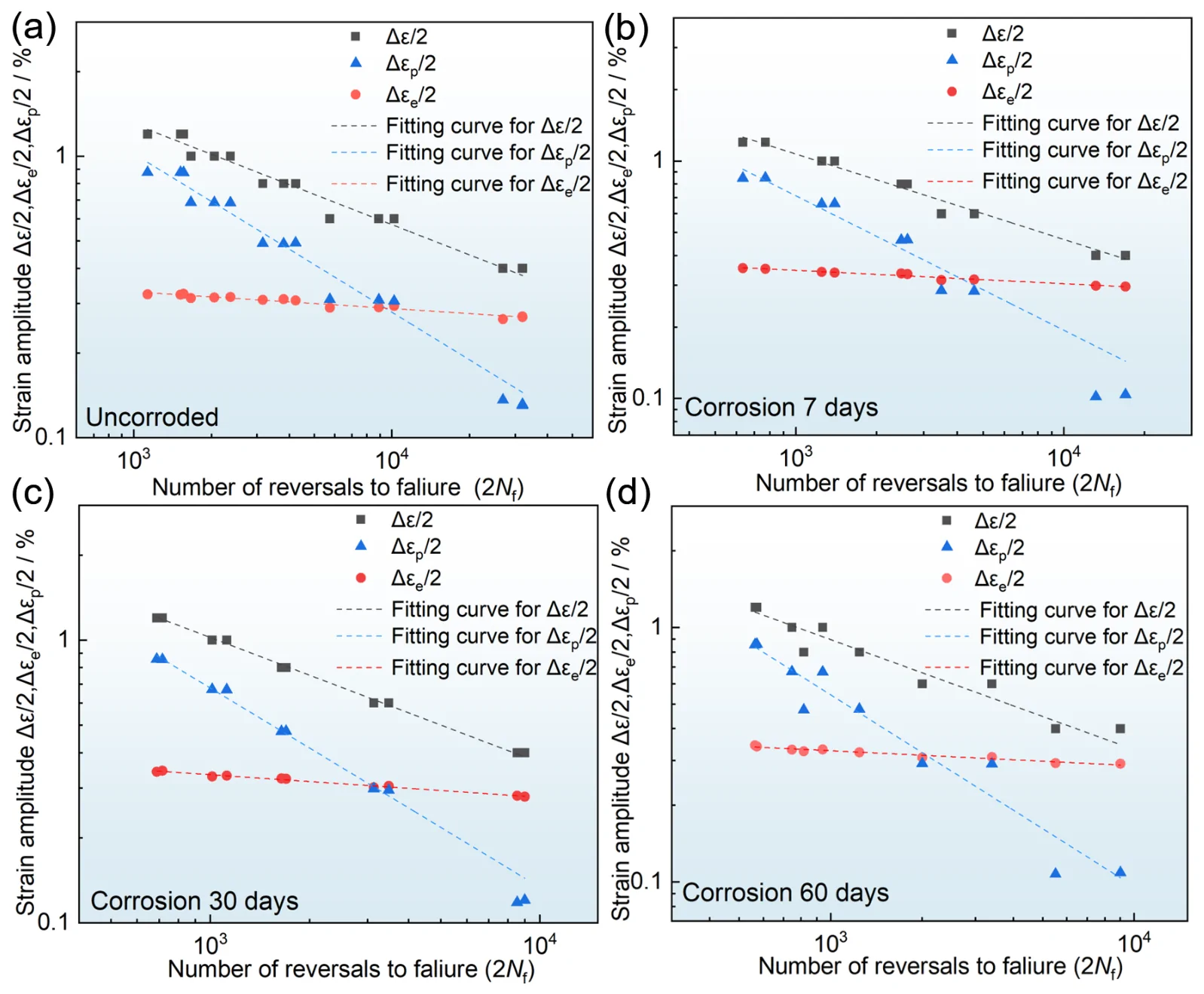
Relationships between elastic, plastic, and total strain amplitudes and fatigue life of Q690qNH steel: (**a**) uncorroded; (**b**) 7 days; (**c**) 30 days; (**d**) 60 days.

Figure 14a–d present the double-logarithmic relationships between the elastic strain amplitude, plastic strain amplitude, total strain amplitude, and the number of reversals to fatigue failure (2*N_f_*) for Q690qNH steel after different corrosion durations. For all corrosion conditions, the strain amplitudes decrease with increasing fatigue life. At relatively low total strain amplitudes, the elastic strain component dominates the fatigue response, whereas with increasing total strain amplitude, the contribution of plastic strain gradually increases and becomes dominant. When the total strain amplitude is approximately 0.6%, the elastic and plastic strain amplitudes are at a comparable level, indicating that the fatigue response of Q690qNH steel within this strain range is governed by the combined contribution of elastic and plastic deformation. With further increase in strain amplitude, the contribution of plastic strain becomes more significant, and the material enters a low-cycle fatigue regime dominated by cyclic plastic deformation.

With increasing corrosion duration, the fitting curves of the elastic and plastic strain amplitudes show only minor variations, while their overall evolution trends remain largely consistent, suggesting that salt-spray corrosion does not substantially alter the elastic–plastic strain-partitioning behavior or the underlying cyclic deformation mechanism of Q690qNH steel. However, compared with the uncorroded specimens, the total strain life curves of the corroded specimens shift toward shorter fatigue lives, indicating that corrosion damage reduces the fatigue life under the same strain amplitude. Therefore, corrosion primarily affects fatigue crack initiation and damage accumulation rather than substantially altering the intrinsic elastic–plastic cyclic deformation mechanism of the material. Combined with the preceding analysis of cyclic stress response and hysteresis behavior, the non-uniform corrosion pits and local defects formed during long-term corrosion may increase stress concentration during cyclic loading and accelerate crack initiation, thereby reducing fatigue life. Nevertheless, owing to the stability of the Q690qNH steel matrix microstructure and cyclic plastic deformation mechanism, the relationship between elastic and plastic strain contributions remains relatively consistent.

### 3.5. Corrosion-Fatigue Crack Initiation and Propagation Mechanism

#### 3.5.1. Crack Initiation and Propagation of the Uncorroded Specimens

**Figure 15:**
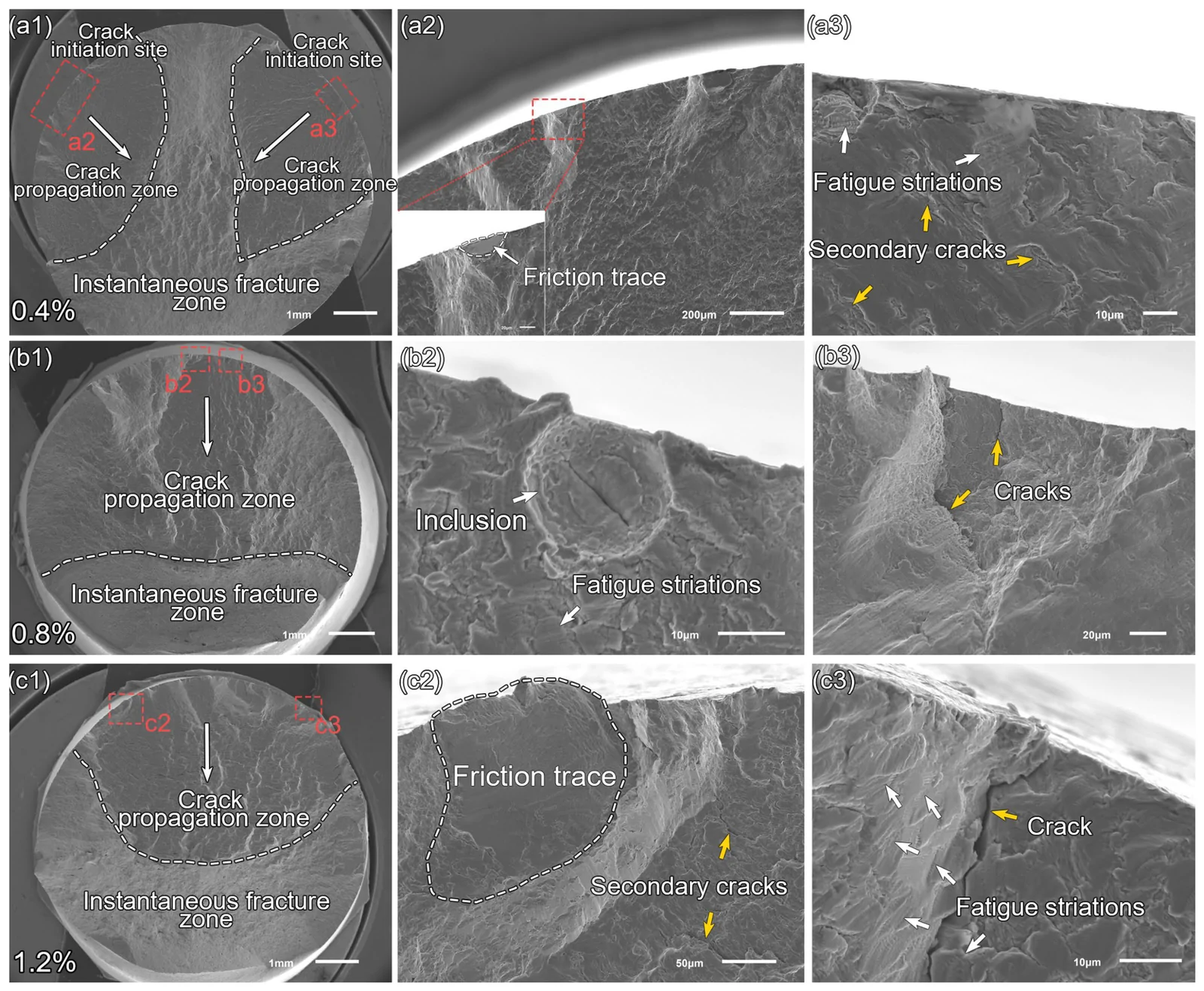
Fatigue fracture morphologies of uncorroded specimens: (**a1**) is the overall view of the fracture surface when the strain amplitude is 0.4%; (**a2**,**a3**) are the magnified views of the initiation sources in (**a1**); (**b1**) is the overall view of the fracture surface when the strain amplitude is 0.8%; (**b2**,**b3**) are the magnified views of the initiation sources in (**b1**); (**c1**) is the overall view of the fracture surface when the strain amplitude is 1.2%; (**c2**,**c3**) are the magnified views of the initiation sources in (**c1**).

To further investigate the low-cycle fatigue fracture mechanisms of Q690qNH steel, scanning electron microscopy (SEM) was employed to characterize the fatigue fracture surfaces of uncorroded specimens subjected to strain amplitudes of 0.4%, 0.8%, and 1.2%. As shown in Figure 15, all low-cycle fatigue cracks initiated at the specimen surface. The fracture surfaces at different strain amplitudes consisted of three characteristic regions: the fatigue crack initiation region (red boxes), the fatigue crack propagation region (white dashed lines), and the final fracture region. At different strain amplitudes, multiple fatigue cracks initiated at different locations, while increasing strain amplitude resulted in larger and more numerous dimples in the final fracture region. At a strain amplitude of 0.4%, fatigue failure was dominated by two major cracks. Numerous undulating ridge-like features were observed at the intersection of the two crack propagation regions, which may have resulted from the coalescence of the two fatigue cracks (Figure 15(a1)). High-magnification observations revealed local friction-induced extrusion marks and accumulated slip bands in the crack initiation region (Figure 15(a2,a3)). Meanwhile, typical fatigue striations and fatigue steps were also observed, indicating that crack initiation was primarily associated with the accumulation of localized slip during cyclic plastic deformation. When the cyclic strain amplitude increased to 0.8%, the cracks still exhibited a multi-site initiation characteristic. Some crack origins were associated with inclusions with surface diameters of approximately 20 μm (Figure 15(b2)), while other regions exhibited distinct fatigue bands and crack propagation features extending from the surface toward the interior (Figure 15(b3)), representing a typical transgranular quasi-cleavage fatigue fracture mode. With a further increase in strain amplitude to 1.2%, the number of crack initiation sites increased significantly, accompanied by the formation of obvious secondary cracks (Figure 15(c1)). Numerous slip traces, friction-induced extrusion regions, and fatigue striations were observed near the crack origins (Figure 15(c2,c3)), indicating that higher cyclic strain promoted surface plastic deformation accumulation and localized damage evolution during crack propagation [47].

Considering the initial microstructural characteristics of Q690qNH steel, the matrix mainly consists of granular bainite, including a ferrite matrix and M-A constituents distributed within it (Figure 2). As indicated by the nanoindentation results presented in Figure 3, the ferritic regions exhibit relatively lower hardness and are therefore more susceptible to localized plastic deformation under cyclic loading. Consequently, the ferrite/M-A constituent interfaces may act as localized strain concentration sites during low-cycle fatigue, facilitating the formation of slip bands and plastic strain accumulation, which further promote fatigue crack initiation [28]. This indicates that fatigue crack initiation in the uncorroded material is primarily governed by the microstructure and cyclic plastic deformation behavior.

**Figure 16:**
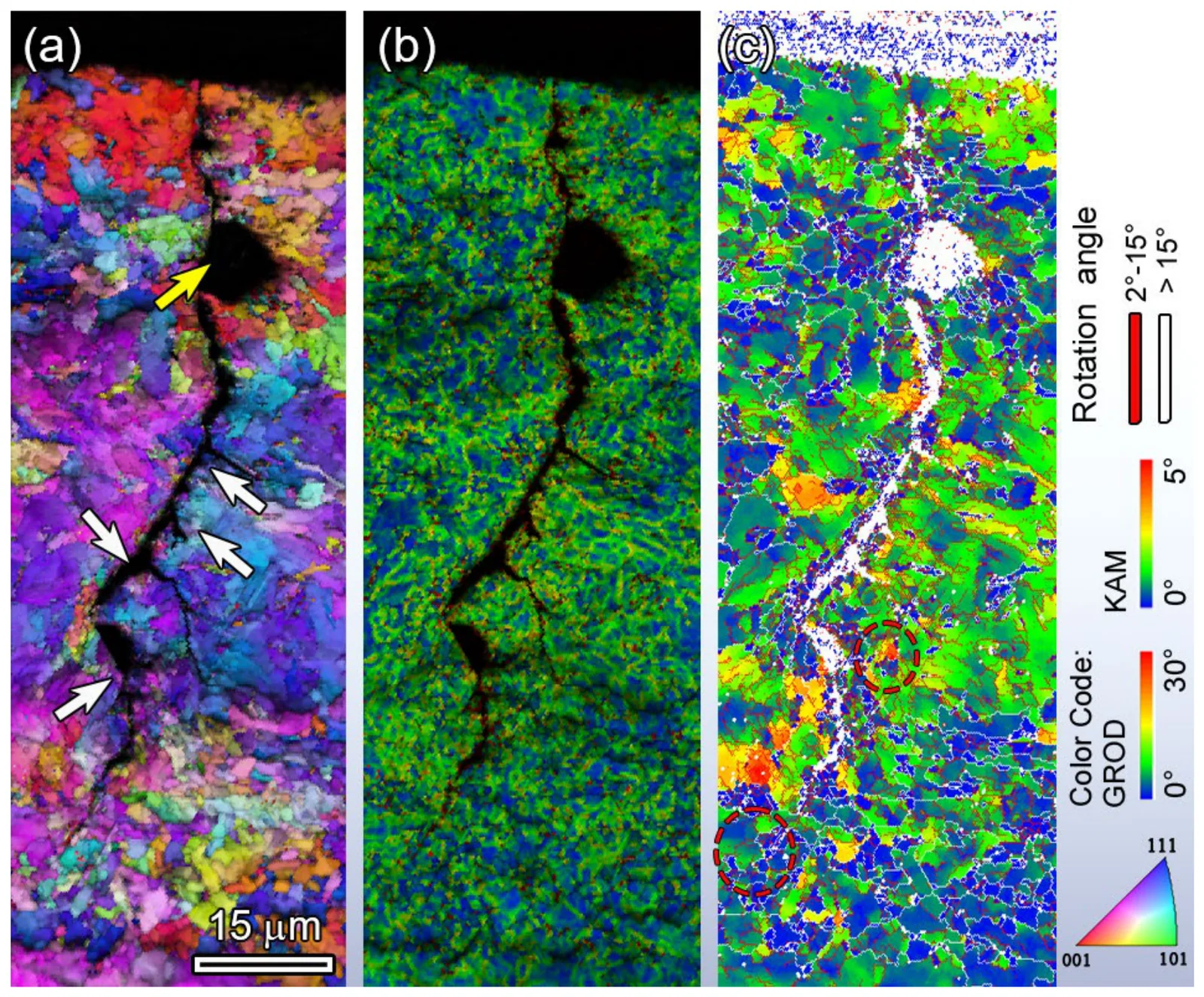
EBSD analysis of secondary cracks in uncorroded fatigue samples: (**a**) IPF superimposed with IQ plots; (**b**) is the KAM plot; (**c**) grain boundary and GROD superposition mapping.

To further elucidate the relationship between crack propagation and microstructural evolution during low-cycle fatigue of Q690qNH steel, EBSD analysis was performed in the crack regions of both uncorroded and corroded specimens, including IPF, KAM, and grain reference orientation deviation (GROD) mappings. For the uncorroded specimen (Figure 16), fatigue cracks initiated at the specimen surface and primarily exhibited a transgranular propagation mode. During propagation, the crack path was deflected by microstructural features such as inclusions, bainitic clusters, and grain-boundary structures. The crack first changed its propagation direction upon encountering an inclusion with a size of approximately 10 μm and subsequently propagated along locally weakened regions. Upon entering the bainitic cluster, obvious crack branching occurred. The KAM and GROD results revealed that the bainitic clusters exhibited high orientation gradients and abundant LAGBs, indicating significant local lattice distortion and localized plastic deformation in these regions. Therefore, the substructures within the bainitic clusters may provide preferential pathways for crack propagation. In contrast, when the crack entered the polygonal ferrite region, its propagation was strongly impeded by HAGBs. The ferrite grains near the crack tip exhibited high GROD values, indicating significant plastic strain accumulation and enhanced local deformation, which contributed to the suppression of crack propagation [48]. Therefore, fatigue crack propagation in uncorroded Q690qNH steel is governed by a competitive process involving localized plastic strain accumulation, microstructure-induced crack deflection, and grain boundary obstruction.

Considering the initial microstructure of the material, granular bainite consists of a ferrite matrix and M-A constituents. Due to the relatively lower hardness of ferrite, it is more susceptible to localized plastic deformation under cyclic tensile-compressive loading. Meanwhile, deformation incompatibility between the M-A constituents and ferrite matrix may promote strain localization at their interfaces. Therefore, ferrite/M-A interfaces may serve as critical regions for fatigue damage accumulation and contribute to early crack initiation.

#### 3.5.2. Corrosion-Assisted Fatigue Crack Initiation and Propagation

**Figure 17:**
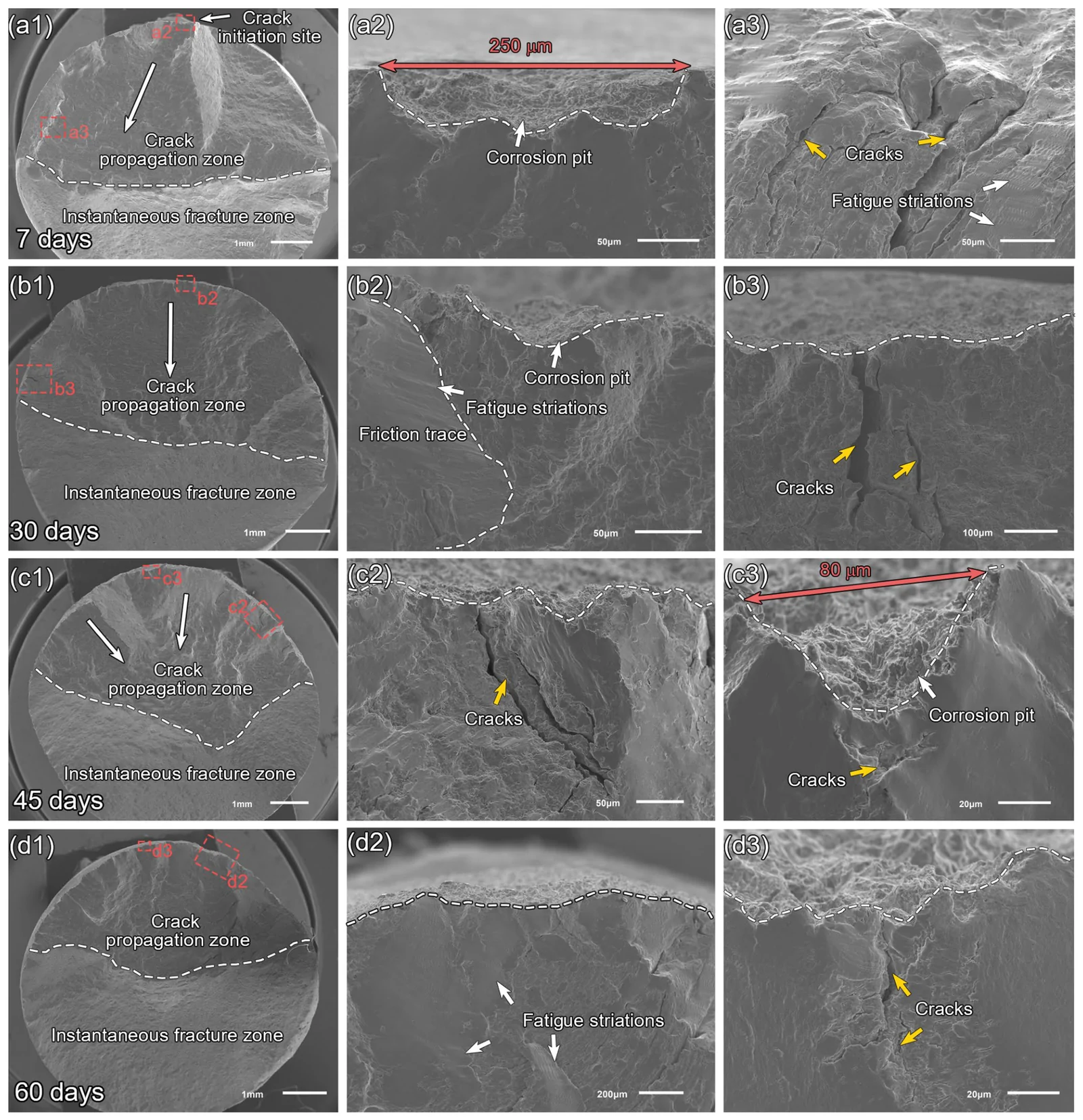
The broken pattern of fatigue samples after strain amplitude of 1.2% corrosion: (**a1**–**a3**) 7 days; (**b1**–**b3**) 30 days; (**c1**–**c3**) 45 days and (**d1**–**d3**) 60 days.

For the corroded specimens, the fatigue fracture mechanism changed markedly, with crack initiation primarily governed by corrosion-induced surface defects. Specimens exposed to different corrosion durations exhibited distinct pit-assisted crack initiation characteristics, demonstrating that surface discontinuities introduced by salt-spray corrosion became critical factors governing the fatigue damage evolution [20,23,49]. After 7 days of corrosion, multiple potential crack initiation sites were observed on the fracture surface (Figure 17(a1)). High-magnification observations revealed that the primary crack origins were associated with a cluster of interconnected small corrosion pits, approximately 250 μm in width and 50 μm in depth (Figure 17(a2)). Cracks extended inward from the pit bottoms, while the propagation regions exhibited river-like quasi-cleavage morphologies accompanied by fatigue striations (Figure 17(a3)). These features indicate that even early-stage corrosion defects were sufficient to alter the preferred sites for fatigue crack initiation. After 30 days of corrosion, the pits developed further and became the predominant crack initiation sites (Figure 17(b1)). During propagation, the interactions and coalescence between adjacent corrosion-damaged regions promoted the formation of continuous main cracks (Figure 17(b2,b3)). This indicates that increasing corrosion severity enhanced interactions among surface defects, thereby accelerating fatigue damage accumulation. After 45 and 60 days of corrosion, macroscopic corrosion pits approaching 1 mm in size appeared on the fracture surfaces (Figure 17(c1,d1)). Although the overall corrosion morphology tended to become more uniform, localized corrosion pits persisted and continued to serve as the primary crack initiation sites. High-magnification observations revealed numerous cracks originating from the pit bottoms, accompanied by fatigue striations and local extrusion marks (Figure 17(c2,c3,d3)), indicating that macroscopic corrosion defects generated during prolonged exposure substantially intensified local stress concentration under cyclic loading.

Therefore, the fatigue crack initiation mechanisms of Q690qNH steel differed substantially before and after corrosion exposure. In the uncorroded material, crack initiation was primarily governed by localized plastic deformation and slip accumulation within the bainitic microstructure, whereas after corrosion, crack initiation gradually became dominated by surface corrosion defects. With increasing corrosion duration, the size and number of corrosion pits increased, causing fatigue crack evolution to transition from localized initiation at isolated defects to a multi-source, cooperative propagation process.

**Figure 18:**
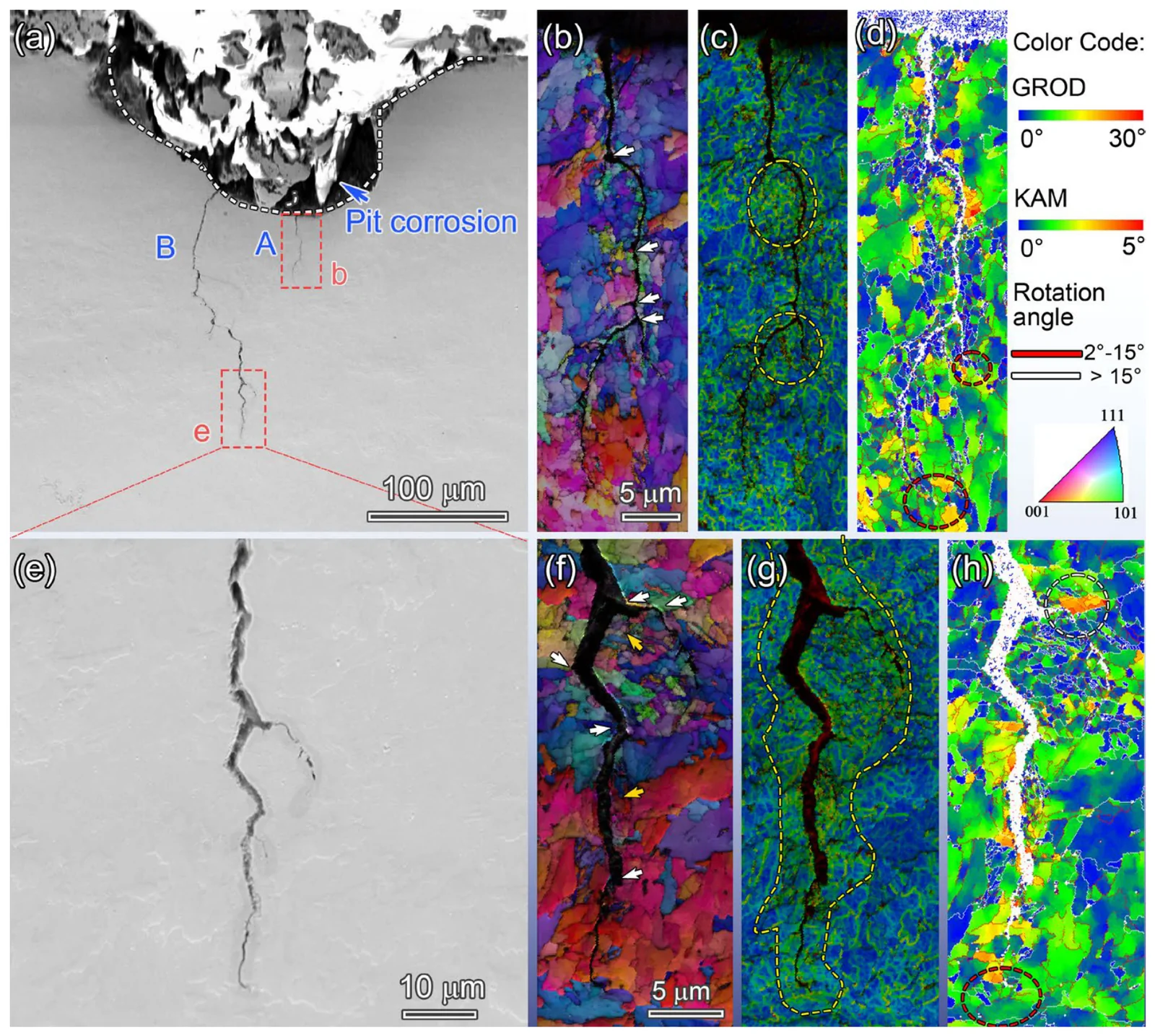
EBSD analysis of secondary cracks of fatigue samples after salt spray corrosion: (**a**,**e**) are SEM images of crack morphology; (**b**,**f**) are IPF superimposed with IQ; (**c**,**g**) are KAM plots; (**d**,**h**) are grain boundaries and GROD diagrams.

For the corroded specimens (Figure 18), fatigue cracks mainly initiated at the bottoms of corrosion pits, indicating that corrosion-induced surface defects replaced microstructural heterogeneities as the dominant crack initiation sites. Taking the 7-day corroded specimen as an example, both cracks initiated from corrosion pits and experienced multiple deflections and branching during propagation. The KAM results revealed pronounced high-KAM regions along the crack paths, indicating severe localized plastic deformation and dislocation accumulation in the vicinity of the corrosion pits. GROD analysis further revealed pronounced orientation gradients near the crack tips, reflecting significant localized cyclic plastic strain. During crack propagation, when encountering bainitic clusters, the cracks were deflected and tended to propagate along low-angle grain boundaries within the bainitic regions. Upon entering ferritic regions, crack propagation was further deflected or temporarily arrested due to the obstruction of HAGBs. This behavior is consistent with that observed in the uncorroded specimens, demonstrating that the matrix microstructure still governs the propagation mode of corrosion fatigue cracks. Nevertheless, corrosion-induced surface defects substantially increased the propensity for crack initiation and promoted localized plastic accumulation, causing cracks to enter the rapid propagation stage earlier [50,51].

Therefore, the effect of corrosion on the low-cycle fatigue performance of Q690qNH steel is manifested primarily during the crack initiation stage, whereas crack propagation remains governed by the combined effects of the bainitic microstructure, grain-boundary characteristics, and localized plastic deformation. The stress concentration introduced by corrosion pits, together with microstructure-controlled crack propagation paths, accelerates fatigue damage evolution, leading to enhanced cyclic softening behavior and reduced fatigue life after corrosion.

## 4. Conclusions

The salt spray corrosion tests were conducted on high-strength weathering steel Q690qNH, followed by low-cycle fatigue tests on both uncorroded and pre-corroded specimens. The effects of pre-corrosion on the low-cycle fatigue performance were evaluated, and the mechanisms of fatigue crack initiation and propagation were analyzed.

(1) Q690qNH weathering steel develops a hierarchical rust layer during cyclic corrosion exposure. The initially formed unstable γ-FeOOH gradually transforms into the more stable α-FeOOH and Fe_3_O_4_ phases, accompanied by an increase in Cr content in the inner rust region. This compositional and structural reconstruction drives the progressive development of a dense, protective inner rust layer, thereby significantly reducing the corrosion rate during prolonged exposure.

(2) Corrosion-induced surface morphological degradation plays a dominant role in deteriorating the low-cycle fatigue performance. The continuous growth and coalescence of corrosion pits intensify local stress concentrations and promote plastic strain localization, providing preferential sites for fatigue crack initiation.

(3) Corrosion damage accelerates cyclic softening and reduces fatigue life. However, as the protective rust layer develops, the rate of fatigue-performance degradation gradually decreases with increasing corrosion exposure time. Thus, under corrosive conditions, the low-cycle fatigue performance of Q690qNH steel is governed by the competition between corrosion-induced defect accumulation and the development of a protective rust layer. However, because only two corroded specimens were tested for each corrosion condition, the apparent deceleration in fatigue degradation should be regarded as a trend and requires further verification through additional replicate tests.

(4) Corrosion exposure alters the fatigue crack initiation mechanism of Q690qNH steel. In uncorroded specimens, fatigue cracks primarily initiate through localized slip accumulation and deformation incompatibility between ferrite and M-A constituents. Following corrosion exposure, corrosion pits progressively replace microstructural heterogeneities as the dominant sites for fatigue crack initiation.

## Figures and Tables

**Table 1:** Chemical compositions (wt.%) of the as-received Q690qNH steel plate.

Materials	Cr	Ni	Cu	Mo	Ti	Al
Q690qNH	<0.6	<1	<0.5	<0.3	<0.02	<0.4
Fe	Nb	C	S	Mn	Si	P
balance	<0.05	0.08	0.003	1.58	0.3	0.012

**Table 2:** Stress concentration factor *K_t_* obtained from tests for Q690qNH steel with each corrosion times.

Specimens	Minimum Size of the Corrosion Pits		Maximum Size of the Corrosion Pits		Average Size of the Corrosion Pits		Stress Concentration Factor *K_t_*
	d (μm)	r (μm)	d (μm)	r (μm)	d (μm)	r (μm)	
Uncorroded	—	—	—	—	—	—	1.000
Cor. 7 days	14.374	32.370	35.846	71.002	25.110	51.686	2.394
Cor. 15 days	20.520	44.900	51.678	98.462	36.099	71.681	2.419
Cor. 30 days	35.290	71.837	88.866	137.103	62.078	104.470	2.542
Cor. 45 days	56.194	101.294	131.454	194.694	93.824	147.994	2.592
Cor. 60 days	80.176	136.084	232.568	317.386	156.372	226.735	2.661

**Table 3:** The fatigue life of Q690qNH steel under each corrosion times and strain amplitude.

Uncorroded		Uncorroded		7 Days		30 Days		45 Days		60 Days	
*N_f_* (Cycles)	Δε/2 (%)	*N_f_* (Cycles)	Δε/2 (%)	*N_f_* (Cycles)	Δε/2 (%)	*N_f_* (Cycles)	Δε/2 (%)	*N_f_* (Cycles)	Δε/2 (%)	*N_f_* (Cycles)	Δε/2 (%)
16016	0.4	1026	1	8488	0.4	4500	0.4	2797	0.4	4506	0.4
15994	0.4	833	1	6581	0.4	4266	0.4	2254	0.4	2754	0.4
13452	0.4	780	1.2	2314	0.6	1740	0.6	1610	0.6	1700	0.6
5099	0.6	759	1.2	1745	0.6	1567	0.6	1465	0.6	1002	0.6
4447	0.6	566	1.2	1305	0.8	849	0.8	808	0.8	736	0.8
2874	0.6			1237	0.8	822	0.8	666	0.8	508	0.8
2118	0.8			698	1	561	1	377	1	471	1
1903	0.8			624	1	507	1	376	1	373	1
1581	0.8			385	1.2	358	1.2	378	1.2	286	1.2
1185	1			317	1.2	344	1.2	246	1.2	282	1.2

**Table 4:** The parameters of Equation (4) for Q690qNH steel with each corrosion times.

Corrosion Times	$\sigma_{f}^{\prime }$/MPa	$\varepsilon_{f}^{\prime }$	b	c	R-Square
Uncorroded	1035	0.6487	−0.0599	−0.5966	0.9806
Cor. 7 days	1058	0.8782	−0.0741	−0.7269	0.9649
Cor. 30 days	980	1.2059	−0.0599	−0.7361	0.9881
Cor. 45 days	949	1.2504	−0.0613	−0.7763	0.9237
Cor. 60 days	918	1.2868	−0.0585	−0.7978	0.9488

## Data Availability

The original contributions presented in this study are included in the article. Further inquiries can be directed to the corresponding author.

## Abbreviations

The following abbreviations are used in this manuscript:

EBSD	electron backscatter diffraction
EDS	energy-dispersive X-ray spectroscopy
FE-SEM	field-emission scanning electron microscope
XRD	X-ray diffraction
3D	three-dimensional
IPF	inverse pole figure
GBs	grain boundaries
IQ	image quality
KAM	kernel average misorientation
GROD	grain reference orientation deviation
HAGB	high-angle grain boundary
LAGB	low-angle grain boundary
LCF	low-cycle fatigue
M-A	martensite-austenite constituent
PAGB	prior austenite grain boundary
SF	softening factor
SR	cyclic strain ratio
